# Evaluation of advanced practice nurse competencies: validation of an instrument for primary care^*^

**DOI:** 10.1590/1518-8345.6958.4449

**Published:** 2025-01-31

**Authors:** Flávia Carvalho Pena Dias, Thelen Daiana Mendonça Ferreira, Maria Silvia Teixeira Giacomasso Vergílio, Pedro Sastre-Fullana, Thaís Moreira São-João, Renata Cristina Gasparino

**Affiliations:** 1 Universidade Estadual de Campinas, Faculdade de Enfermagem, Campinas, SP, Brazil.; 2 Scholarship holder at the Coordenação de Aperfeiçoamento de Pessoal de Nível Superior (CAPES), Brazil.; 3 Universidade Estadual de Campinas, Grupo de Estudos e Pesquisa em Tecnologias para Gestão em Enfermagem (GEPTGE), Campinas, SP, Brazil.; 4 Hospital Universitari Son Espases, Palma de Mallorca, Illes Balears, PM, Spain.; 5 University of Rhode Island, College of Nursing, Kingston, RI, United States of America.

**Keywords:** Validation Study, Reproducibility of Results, Advanced Practice Nursing, Professional Competence, Nurses, Primary Health Care

## Abstract

**Objective:**

to evaluate the construct validity and reliability of the Brazilian version of the Instrument for the Evaluation of Advanced Practice Nurse Competencies in Primary Health Care.

**Method:**

methodological study carried out in 78 primary health care centers in the southeast of Brazil, with 215 nurses. Data was collected using three instruments: the sample characterization form, the Brazilian version of the Advanced Practice Nurse Competency Assessment Instrument and the Therapeutic Interventions category of the Nurse Competency Scale. Construct validity was verified using confirmatory factor analysis and Spearman’s correlation coefficient. Reliability was assessed using Cronbach’s alpha and composite reliability.

**Results:**

in the factor analysis, the model converged on a satisfactory result, with six items excluded, resulting in an instrument with 38 items distributed over eight dimensions. In convergent construct validity, positive, significant correlations were observed (p<0.0001) and ranged from weak to moderate in magnitude (r=0.2701 and r=0.397). Satisfactory evidence was found through the Composite Reliability analysis (0.78-0.89).

**Conclusion:**

the instrument showed evidence of construct validity and internal consistency. It could be used to help implement strategies for developing advanced practice in nursing.

## Highlights

**(1)** Encourage discussion on Advanced Practice Nursing (APN) in Brazil.

**(2)** To provide an instrument for evaluating APN in Primary Health Care.

**(3)** To help implement APN in Brazil in Primary Health Care.

**(4)** The Brazilian version of the instrument can be applied in different contexts.

**(5)** A strategy for strengthening the nursing workforce.

## Introduction

The worldwide implementation of Advanced Practice Nursing (APN) has demonstrated a positive and progressive impact on the quality of care, clinical outcomes and coverage of health services^(1-2)^. In addition, it is seen as an important strategy for recognizing^(3)^ and strengthening the workforce, including the qualification, recruitment and retention of nurses^(4)^, which contributes to reducing costs for healthcare institutions^(1-2,5)^.

The United States and Canada were the great exponents of this practice and after the 1960s, there was a rapid expansion of APN to other continents^(6)^. Currently, different countries are working to reformulate the regulation of the scope of practice^(7)^.

The World Health Organization (WHO), together with the Pan American Health Organization (PAHO), have been encouraging the implementation of APN in Latin America and the Caribbean, especially in Primary Health Care (PHC), in view of the need to expand care in remote regions and increase coverage of access to health services^(4,8)^.

In this context, in 2018, PAHO published the document “Expanding the Role of Nurses in Primary Health Care”^(8)^, and in 2020, WHO published the alert “Urgent Health Challenges for the Next Decade”, highlighting priorities such as fairer access to health services, preparing for epidemics, guaranteeing access to medicines, and strategies for investing in professionals who protect the health of the population^(9)^.

In Brazil, a discussion on the “Implementation of the APN” began in 2015, promoted by the Federal Nursing Council (Cofen) and the Brazilian Nursing Association (ABEn)^(10)^. However, this practice, which is fundamental for guaranteeing the quality of care, is still in its infancy^(11)^.

The APN must have a specialized knowledge base and the skills to carry out assessments, propose diagnoses and prescriptions, implement programs and care plans and act as a reference for users and health services^(12)^. In addition, they must have a master’s degree, be part of multi-professional teams and contribute to care management^(8,12)^.

Therefore, the standards and competencies of APNs are being developed and adapted to meet the needs of each country in relation to the training and continuing education of professionals in health services^(13)^. As such, researchers have been mapping out a profile of essential competencies to support the definition of the scope of this practice^(14)^.

In this context, the *Inventario para la Evaluación de Competencias en Enfermeras de Práctica Avanzada* - IECEPA, developed in Spain, is considered to be an instrument with evidence of validity and reliability for assessing APN competencies, and is applicable to any level of health care, especially PHC^(15)^.

In 2022, this instrument was adapted for Brazilian culture and entitled the Instrument for the Evaluation of Advanced Practice Nurse Competencies - IECEPA Brazilian version. Its content was evaluated and showed evidence of validity^(16)^.

According to the literature, there are instruments for assessing APN competencies. Although three have been built and/or adapted for Brazilian Portuguese, there are still no scientific publications on the evaluation of the reliability and validity of these instruments^(13-18)^. It is important to use instruments with evidence of reliability and validity in order to properly assess the competencies of nurses in advanced practice.

The application of the tool in question in PHC is vast and promising, as it will make it possible to map APN competencies and implement interventions that could offer tangible benefits for clinical practice, professional development and the advancement of research in the field of nursing, and could innovate and reform health systems to respond to the problems arising from the needs of the population, especially given the increase in people with chronic conditions being monitored in PHC^(2,10)^. Therefore, this study aimed to assess the validity and reliability of the Brazilian version of the Instrument for the Evaluation of Advanced Practice Nurse Competencies in Primary Health Care^(15-16)^.

## Method

### Study design

This methodological study aimed to analyze the construct validity (structural and hypothesis testing) and reliability (internal consistency)^(19)^ of the IECEPA Brazilian version^(16)^, using the criteria adopted from the COnsensus-based Standards for the selection of health Measurement INstruments (COSMIN) checklist^(19)^.

### Place of data collection

Data was collected from 78 PHC centers in two municipalities in the southeast region. City “A” had 1,194,094 inhabitants, 64 Primary Health Centers (PHC), most of which included the Family Health Strategy (FHS), 291 nurses and five geographical areas, each with approximately 200,000 inhabitants. City “B” had 129,193 inhabitants, 14 PHC without the presence of the PSF at the time of collection, and 17 nurses, with around 9,228 inhabitants for each center.

### Period

The data collection stage took place between August 2020 and July 2021, with face-to-face data collection between August and October 2020 and hybrid data collection between October 2020 and July 2021 due to the COVID-19 pandemic.

### Participants and selection criteria

To estimate the sample size, the internationally recommended criterion for factor analysis was adopted, which considers a minimum of 100 participants and five respondents for each item of the instrument to be appropriate^(19)^, resulting in a sample of 220 professionals.

As a result, 256 nurses were approached for convenience; however, 41 (16%) were excluded (31 did not hand in the questionnaire within the agreed timeframe and 10 handed in the questionnaire incompletely). Thus, the final sample consisted of 215 participants (59 approached online and 156 in person).

Based on these criteria, all active nurses were selected for convenience and those who did not complete one or more items on the Brazilian version of the IECEPA were excluded.

### Instruments used to collect information

#### Personal and Professional Characterization Form

Personal data (age, gender and marital status) and professional data (length of experience as a nurse, length of experience in the area in which they worked and training) were collected.

#### IECEPA Brazilian version^(16)^

The IECEPA Brazilian version was designed to assess the competence of nurses according to the necessary roles and standards of APN in primary care. This instrument has 44 items divided into eight dimensions: Research and Evidence-Based Practice: promotes the relationship between research, identification of scientific evidence and clinical practice (1.1-1.8); Clinical and Professional Leadership: demonstration of leadership in promoting quality care and providing advice to other health professionals (2.1-2.4); Professional Autonomy: assessment of autonomy in the use of pharmacological and non-pharmacological interventions, clinical diagnosis and referral to other professionals (3. 1-3.8); Interprofessional Relations and Mentoring: reflects the ability to collaborate with other professionals to improve care for the user and serve as a clinical reference (4.1-4.6); Quality Management: based on skills to evaluate and promote the quality and effectiveness of advanced care, identifying and acting on problems related to quality and patient safety (5.1-5.4); Care Management: represents the coordination of care at all levels of the health system (6.1-6.6); Teaching and Professional Education: reflects the role of educator, promoting an environment conducive to learning for users, family members, nurses, students and other health professionals (7.1-7.4) and Health Promotion: focuses on improving or recovering the health of the user, regardless of the context (8.1-8.4)^(15-16)^.

These items are assessed using a five-point Likert scale, ranging from never (one point) to always (five points); the higher the score, the more frequently the competency described is used in daily professional activities. The average scores of the participants’ responses are calculated to obtain the dimension scores^(15-16)^.

#### Nurse Competence Scale (NSC)^(20)^

The Nurse Competence Scale (NSC) is a tool that assesses how often a nurse uses a particular competence. It has 73 items divided into seven categories. In this study, only the Therapeutic Interventions category was used (α=0.87), which has 10 items (39, 40, 41, 42, 43, 44, 45, 46, 47, 48), which reflect the dimensions of the IECEPA Brazilian version. The response scale for each item is Likert-type with four options ranging from zero (“does not apply to my practice”) to three (“used very frequently”). The higher the score, the more frequently the professional uses that skill in their professional activities^(20)^. The Therapeutic Interventions category of the NCS was used to assess construct validity by testing the following hypothesis: the higher the score in the dimensions of the Brazilian version of the IECEPA, the higher the score in the Therapeutic Interventions category of the NCS.

### Data collection

Data collection was carried out in a hybrid way due to the pandemic, with face-to-face (in situ) and online recruitment, through institutional emails sent by the health departments of the participating cities, with three waves of collection. The nurses were approached at their workplaces, the purpose of the study was explained, and after obtaining their consent and signing the Informed Consent Form (ICF), they completed the printed instruments.

### Data processing and analysis

The data collected was entered into the Excel for Windows/XP^®^ program and transferred to Statistical Analysis Software^®^ version 9.4 and Smart Partial Least Squares (PLS) 3.2.1.^®^ to carry out the analyses. Initially, descriptive statistics, absolute and relative frequencies of categorical variables and measures of position (mean, minimum, maximum) and dispersion (standard deviation) of continuous variables were used.

To analyze the measurement properties of the IECEPA Brazilian version, a factor analysis was carried out using structural equation models with PLS^(21)^, in which APN competencies were considered a second-order variable.

In evaluating the model, the Average Variance Extracted (AVE) was calculated for each dimension and values above 0.5 indicated that the model presented a satisfactory result^(22)^. The cross-loadings were examined to determine whether the factor loading of an item was higher in the factor in which it was originally allocated; the discriminant validity of the model was checked using the Fornell-Larcker criterion, which analyzed whether the square roots of the AVE were greater than the correlations between the dimensions^(23)^.

To test the hypothesis, the Shapiro-Wilk test was used to evaluate the distribution of the data and the Spearman coefficient^(24)^ was used to correlate the scores in the “Therapeutic Interventions” category of the NCS with each of the dimensions of the IECEPA Brazilian version. The values were classified as weak (0.1 to 0.29), moderate (0.3 to 0.49) or strong (0.5)^(25)^. A significance level of 5% (p <0.05) was used for all tests^(24)^.

Reliability was assessed by means of internal consistency and, for this, Cronbach’s alpha coefficient and Composite Reliability (CC) were calculated, where values equal to or greater than 0.70 were considered acceptable^(24)^.

For acceptability, values equal to or greater than 80% were considered satisfactory^(26)^.

### Ethical aspects

Before carrying out the study, authorization was obtained from the author of the original instrument. The project was approved on March 26, 2020 (in-person format) and October 21, 2020 (online format) by the institution’s Research Ethics Committee with the Certificate of Submission for Ethical Appraisal No. 23193319.0.0000.5404 (Opinions 3.936.606 and 4.351.709, respectively) and complied with the ethical recommendations regarding research carried out with human beings following Resolution 466/2012 of the National Health Council.

## Results

With regard to sociodemographic characteristics, the average age of the 215 nurses was 39.0 years (SD=7.8), with a predominance of women (n=186; 86.5%) and married people (n=124; 57.6%). With regard to professional characterization, the average length of experience in the profession was 12.3 years (SD=7.6) and in the area in which they worked at the time of data collection was 9.0 years (SD=7.7). The majority had a postgraduate specialization degree (n=124; 57.6%) and 88 (40.9%) had a postgraduate degree in Public Health. Regarding the assessment of acceptability, of the 256 nurses initially approached, 215 (83.9%) participants answered all the items on the IECEPA Brazilian version for PHC, with 31 (12.0%) nurses not answering, possibly due to the work overload caused by the COVID-19 pandemic, as well as 10 (4.0%) answering incompletely.

In the initial analyses of the instrument’s structure, the model revealed AVE values of less than 0.50 for four dimensions, as can be seen in Table 1.

**Table 1:** - Initial model of the average variance extracted, composite reliability and Cronbach’s alpha of the dimensions of the Instrument for Assessing the Competencies of Advanced Practice Nurses Brazilian version for primary health care (n = 215). Campinas, SP, Brazil, 2020-2021

Dimensions of the IECEPA* Brazilian version		**Initial model**	
	**AVE** ^†^	**Composite Reliability**	**Cronbach’s alpha**
1 - Research and Evidence-Based Practice	*0.42*	0.85	0.81
2 - Clinical and Professional Leadership	*0.47*	0.78	*0.64*
3 - Professional Autonomy	0.51	0.89	0.86
4 - Interprofessional Relations and Mentoring	*0.41*	0.81	0.71
5 - Quality Management	0.56	0.83	0.74
6 - Care Management	*0.47*	0.84	0.77
7 - Teaching and Professional Education	0.62	0.87	0.79
8 - Health Promotion	0.54	0.82	0.71

*IECEPA = Instrument for the Evaluation of Advanced Practice Nurse Competencies Brazilian version; ^†^AVE = Average Variance Extracted

After evaluating the SEM, the cross-factor loadings of the instrument items were analyzed, which led to the exclusion of items 1.1 from the Research dimension, 2.3 from the Leadership dimension, 4.6 from the Relationships dimension and 6.1 from the Care Management dimension, as they had the lowest factor loadings in their respective dimensions.

Despite the exclusion of four items, the AVE of the Research and Relationships dimensions did not reach the established value (0.45 and 0.46, respectively) and so items 1.2 of the Research dimension and 4.1 of the Relationships dimension were excluded. Table 2 shows the values achieved for the SEM, CC and Cronbach’s alpha for each dimension of the IECEPA Brazilian version, after excluding the six items (1.1, 1.2, 2.3, 4.1, 4.6 and 6.1).

**Table 2:** - Final model of the average variance extracted, composite reliability and Cronbach’s alpha of the dimensions of the Instrument for Assessing the Competencies of Advanced Practice Nurses Brazilian version for primary health care (n = 215). Campinas, SP, Brazil, 2020-2021

Dimensions of the IECEPA* Brazilian version		**Initial model**	
	**AVE** ^†^	**Composite Reliability**	**Cronbach’s alpha**
1 - Research and Evidence-Based Practice	*0.48*	0.85	0.79
2 - Clinical and Professional Leadership	0.54	0.78	*0.57*
3 - Professional Autonomy	0.51	0.89	0.86
4 - Interprofessional Relations	0.52	0.81	0.68
5 - Quality Management	0.56	0.83	0.74
6 - Care Management	0.51	0.84	0.76
7 - Teaching and Professional Education	0.62	0.87	0.79
8 - Health Promotion	0.55	0.82	0.71

*IECEPA = Instrument for the Evaluation of Advanced Practice Nurse Competencies Brazilian version; ^†^AVE = Average Variance Extracted

After excluding the items, the final model showed that the AVE value for the Research dimension did not reach the established value. Thus, the item with the lowest factor loading was item 1.3 “I identify research priorities in my area of professional practice” (0.61) and had a CC and Cronbach’s alpha above the established value. In view of this, the researchers decided to consult the author of the original instrument before excluding this item. The author recommended the item to be kept due to its conceptual importance, despite the fact that the EMV of this dimension was below the established level.

The factor loadings of the items in their respective dimensions and cross-factor loadings are shown in Table 3.

**Table 3:** - Factor loadings of the items in their respective constructs (highlighted) and cross-factor loadings (n = 215). Campinas, SP, Brazil, 2020-2021

**Item**	D1*	**D2** ^†^	**D3** ^‡^	**D4** ^§^	**D5** ^||^	**D6** ^¶^	D7**	**D8** ^††^
1.3	*0.61*	0.26	0.05	0.10	0.31	0.22	0.05	0.03
1.4	*0.65*	0.29	0.37	0.24	0.25	0.28	0.09	0.36
1.5	*0.72*	0.37	0.21	0.28	0.32	0.31	0.24	0.24
1.6	*0.75*	0.30	0.19	0.24	0.39	0.31	0.15	0.18
1.7	*0.73*	0.31	0.13	0.11	0.38	0.22	0.05	0.16
1.8	*0.69*	0.41	0.20	0.31	0.32	0.30	0.17	0.26
2.1	0.29	*0.83*	0.23	0.29	0.32	0.24	0.16	0.28
2.2	0.35	*0.77*	0.08	0.23	0.35	0.17	0.10	0.20
2.4	0.44	*0.59*	0.16	0.14	0.26	0.04	0.16	0.12
3.1	0.10	0.21	*0.62*	0.18	-0.01	0.09	0.23	0.37
3.2	0.18	0.21	*0.75*	0.29	0.19	0.23	0.17	0.37
3.3	0.15	0.19	*0.73*	0.25	0.21	0.22	0.25	0.46
3.4	0.36	0.21	*0.74*	0.29	0.25	0.30	0.21	0.48
3.5	0.26	0.16	*0.79*	0.37	0.16	0.40	0.24	0.47
3.6	0.19	0.09	*0.73*	0.23	0.17	0.23	0.14	0.38
3.7	0.10	0.04	*0.73*	0.28	0.04	0.32	0.22	0.46
3.8	0.27	0.14	*0.61*	0.29	0.17	0.24	0.14	0.34
4.2	0.33	0.22	0.27	*0.71*	0.31	0.31	0.22	0.19
4.3	0.19	0.20	0.27	*0.79*	0.32	0.35	0.32	0.33
4.4	0.19	0.17	0.34	*0.64*	0.18	0.27	0.22	0.28
4.5	0.23	0.30	0.24	*0.72*	0.33	0.35	0.23	0.19
5.1	0.28	0.20	0.07	0.27	*0.69*	0.32	0.11	0.19
5.2	0.32	0.30	0.23	0.33	*0.79*	0.40	0.23	0.26
5.3	0.43	0.37	0.28	0.35	*0.81*	0.45	0.17	0.31
5.4	0.36	0.36	0.00	0.23	*0.69*	0.30	0.15	0.19
6.2	0.21	0.14	0.22	0.35	0.38	*0.64*	0.27	0.27
6.3	0.24	0.21	0.33	0.34	0.21	*0.70*	0.25	0.44
6.4	0.36	0.16	0.30	0.34	0.40	*0.83*	0.27	0.42
6.5	0.33	0.16	0.23	0.28	0.41	*0.73*	0.33	0.36
6.6	0.28	0.11	0.22	0.30	0.39	*0.68*	0.42	0.36
7.1	0.14	0.12	0.26	0.28	0.14	0.35	*0.80*	0.35
7.2	0.10	0.16	0.22	0.25	0.18	0.29	*0.79*	0.38
7.3	0.23	0.17	0.19	0.32	0.27	0.42	*0.80*	0.38
7.4	0.12	0.16	0.20	0.23	0.11	0.28	*0.76*	0.33
8.1	0.30	0.15	0.31	0.29	0.32	0.37	0.26	*0.55*
8.2	0.23	0.19	0.44	0.23	0.23	0.31	0.30	*0.74*
8.3	0.17	0.20	0.52	0.29	0.15	0.36	0.33	*0.80*
8.4	0.25	0.27	0.45	0.23	0.28	0.48	0.45	*0,83*

*D1 = Dimension 1 (Research and Evidence-Based Practice); ^†^D2 = Dimension 2 (Clinical and Professional Leadership); ^‡^D3 = Dimension 3 (Professional Autonomy); ^§^D4 = Dimension 4 (Interprofessional Relations); ^||^D5 = Dimension 5 (Quality Management); ^¶^D6 = Dimension 6 (Care Management); **D7 = Dimension 7 (Teaching and Professional Education); ^††^D8 = Dimension 8 (Health Promotion)

In order to assess discriminant validity (Table 4), the square roots of the SMVs, which were highest in the factor in which they were initially allocated, were checked.

**Table 4:** - Discriminant validity of the factor model, according to the Fornell-Larcker criterion of the Instrument for the Evaluation of Advanced Practice Nurse Competencies Brazilian version (n = 215). Campinas, SP, Brazil, 2020-2021

**Dimensions of the IECEPA** ^*^ **Brazilian version**	**1**	**2**	**3**	**4**	**5**	**6**	**7**	**8**
1- Evidence-based Research and Practice	*0.69*							
2- Clinical and Professional Leadership	0.47	*0.74*						
3 - Professional Autonomy	0.29	0.22	*0.71*					
4 - Interprofessional Relations	0.33	0.31	0.39	*0.72*				
5 - Quality Management	0.47	0.42	0.22	0.40	*0.75*			
6 - Care Management	0.40	022	0.37	0.45	0.50	*0.72*		
7- Teaching and Professional Education	0.19	0.19	0.28	0.35	0.23	0.43	*0.79*	
8 - Health Promotion	0.32	0.28	0.59	0.35	0.33	0.52	0.48	*0.74*

*IECEPA = Instrument for the Evaluation of Advanced Practice Nurse Competencies Brazilian version

To assess the convergent construct validity, the correlations between each dimension of the IECEPA, Brazilian version, and the Therapeutic Interventions category of the NCS were tested (Table 5).

**Table 5:** - Correlation between the dimensions of the Brazilian version of the Advanced Practice Nurse Competency assessment tool and the therapeutic interventions category of the nurse competency scale (n = 215). Campinas, SP, Brazil, 2020-2021

Dimensions of the IECEPA* Brazilian version	**Therapeutic Interventions ECE** ^†^
1 - Research and Evidence-Based Practice	0.3888 *<0.0001* ^‡^
2 - Clinical and Professional Leadership	0.3508 *<0.0001* ^‡^
3 - Professional Autonomy	0.2701 *<0.0001* ^‡^
4 - Interprofessional Relations	0.3555 *<0.0001* ^‡^
5 - Quality Management	0.3555 *<0.0001* ^‡^
6 - Care Management	0.3972 *<0.0001* ^‡^
7 - Teaching and Professional Education	0.3419 *<0.0001* ^‡^
8 - Health Promotion	0.3049 *<0.0001* ^‡^

*IECEPA = Instrument for the Evaluation of Advanced Practice Nurse Competencies Brazilian version; ^†^NCS = Nurse Competency Scale; ^‡^p-value = Obtained using Spearman’s correlation coefficient

## Discussion

The aim of this study was to evaluate the construct validity and reliability of the Brazilian version of the IECEPA in PHC. According to researchers, APS competencies should be widely promoted, given the need to develop complex skills, both in clinical practice and in management, education and research^(11,13)^.

To this end, measurement instruments must include a rigorous analysis of the constructs they are intended to measure in order to be used safely in clinical practice and research^(27)^. The use of validated instruments to map APN competencies is necessary to boost the implementation and improvement of the scope of this practice^(11)^. Studies show that APNs in PHCs increase users’ access to health services while improving health systems’ ability to respond to challenges such as socioeconomic, epidemiological, and climatic changes through updated health policies and actions^(1-2,10)^.

The data obtained in this study suggests that the instrument was satisfactorily accepted by the target audience^(26)^, which was reflected in the acceptance of the instrument by the respondents, since the majority of participants answered all the items. This acceptability is in line with other studies^(28-29)^.

Regarding the measurement properties, the results showed that in the evaluation of internal consistency, the Cronbach’s alpha of the dimensions (0.57 - 0.86) was lower than that of the original study (0.81 - 0.92)^(15)^. These changes may be related to the characteristics of the participating sample, the context and the timing of the pandemic^(27)^. However, the WC, a more robust measure of internal consistency, showed satisfactory reliability of the dimensions of the Brazilian version of the instrument^(17)^.

In terms of structural construct validity, it was found that, in the confirmatory factor analysis, six items from four dimensions were excluded in order to better adjust the AVE. In dimension 1 - Research, it was found that even with the exclusion of two items, the AVE was lower than the established value. A probable explanation for this result is that this dimension is not used as much in the practice of the nurses participating in this research, due to the lack of investment in research, the high demand for care and/or the lack of support from management^(7)^. These data provide an opportunity to discuss the incorporation of the APN as a public policy that supports managers in a number of ways, including the implementation of evidence-based practice in the context of PHC, with the aim of further improving the quality of health services and the effectiveness of interventions for the population served^(7,10-11)^.

Regarding item 4.6, it was noted that it was the only one that mentioned the word “mentoring”. This prompted an additional consultation with experts, who agreed to remove the word “mentoring” from the name of the fourth dimension, which was renamed Interprofessional Relationships. Some studies have also made these changes to the structure of the instrument to make it more suitable for research^(11,28-30)^.

Another important aspect to note is that the discriminant validity of the model was analyzed using cross-loadings and the Fornell-Larcker criterion, showing that the constructs were independent and that the items were accurate in measuring the dimensions in which they were initially allocated^(22)^.

In order to assess structural construct validity, a structural equation model was used with the PLS estimation method, which has the advantages of being able to build more complex models and the absence of the assumption that the variables included in the analysis have a normal distribution^(20)^. Although it is a relatively new methodology in the scientific literature compared to the traditional method of analysis of covariance (CB-SEM), PLS has been a valuable option in various studies^(28-30)^.

At the end of the analysis, it was found that the Brazilian version applied in PHC had a modified structure compared to the original version^(15)^, with eight dimensions and 38 items. This variation in the structure of the instrument is a strategy to improve the measurement properties and take into account cultural differences between countries^(31)^. In addition, researchers refer to the use of international instruments to assess the competence of APNs and describe nurses as those who have the clinical competence to work in PHC^(1-2,10)^.

With regard to the results of the convergent construct validity analysis, the hypothesis formulated was confirmed, showing positive and significant correlations between all the dimensions of the Brazilian version of the IECEPA and the Therapeutic Interventions category of the NCS. In seven subscales, the magnitude of the correlation was considered moderate, which suggests that an increase in APN competence is associated with a greater ability to carry out therapeutic interventions in everyday life. Studies^(28,30)^ corroborate similar results regarding the same strength of correlation.

In view of this, the feasibility of the IECEPA Brazilian version in PHC will provide a contribution to the national literature, collaborating in the revision of pedagogical projects and the implementation of postgraduate courses and in the development of competencies necessary for the expanded role of nurses. In addition, the results of these studies may be useful for the development of public policies to improve the quality of health care offered to the Brazilian population.

In summary, nurses’ practice in PHC can be improved through the implementation of APS models, offering advantages such as increased access and comprehensive care, but also facing challenges such as professional resistance and legislative barriers. The success of these models depends on investments in training, adequate supervision and overcoming regulatory obstacles^(10,17)^.

Although there are limitations in the application of self-assessment measures which include the exclusion of missing cases, memory bias, social desirability and cognition of understanding, this method is constantly used by researchers due to its economic benefit, reflections of the past and practicality.

With regard to the 16% of missing data, it was decided not to impute the missing or absent information in order to ensure the reliability and validity of the analyses^(32)^, promoting sound scientific research, since imputation techniques can introduce potentially inaccurate data, cause bias or distort statistical properties.

The use of a hybrid data collection approach has several significant advantages, including methodological flexibility, allowing different data collection methods to be combined. This adaptive flexibility is especially relevant in contexts where physical restrictions, such as those imposed by the COVID-19 pandemic, can impact the conduct of face-to-face interviews and conventional personal approaches^(33)^.

Although this survey covered all PHC services in the two cities, it was not possible to obtain the desired feedback, which limits the generalizability of the results. In addition, some nurses who could meet the criteria for defining APN may not have been reached, due to the convenience survey approach and the conditions resulting from the pandemic.

It should be noted that more studies need to be carried out in different contexts and populations to confirm the properties of the instrument, as well as cross-sectional and correlational studies and clinical trials. However, the validation of the Brazilian version of the IECEPA was rigorous in its methodological phases and statistical analysis.

## Conclusion

The IECEPA Brazilian version showed evidence of construct validity and internal consistency in the sample studied. Therefore, the contributions of this study include advances in the validation of assessment instruments, recognition of the advanced role of nurses and the urgent need to improve training and encourage evidence-based research and practice. In addition, it highlights the improvement of access and quality of care, as well as support for the development of more effective health policies in PHC.

It highlights the need to negotiate the expansion of practice and the creation of legislation that supports the professional, ensuring that nurses can act fully in care according to their professional training, thus contributing to the fulfillment of health policies. Its future application will make it possible to map out and implement strategies for developing APNs in PHC.
