# Supplementary material for: Evaluation of advanced practice nurse competencies: validation of an instrument for primary care
Source: Rev Lat Am Enfermagem. 2025 Jan 31;33:e4449. doi: 10.1590/1518-8345.6958.4449 (PMC11789756; doi:10.1590/1518-8345.6958.4449)
Supplement: Translation

# Translation: Evaluación de competencias del enfermero de práctica avanzada: validación de un instrumento para atención primaria^*^

**DOI:** 10.1590/1518-8345.6958.4448

**Authors:** Flávia Carvalho Pena Dias, Thelen Daiana Mendonça Ferreira, Maria Silvia Teixeira Giacomasso Vergílio, Pedro Sastre-Fullana, Thaís Moreira São-João, Renata Cristina Gasparino

**Affiliations:** 1 Universidade Estadual de Campinas, Faculdade de Enfermagem, Campinas, SP, Brasil.; 2 Becaria de la Coordenação de Aperfeiçoamento de Pessoal de Nível Superior (CAPES), Brasil.; 3 Universidade Estadual de Campinas, Grupo de Estudos e Pesquisa em Tecnologias para Gestão em Enfermagem (GEPTGE), Campinas, SP, Brasil.; 4 Hospital Universitari Son Espases, Palma de Mallorca, Illes Balears, PM, España.; 5 University of Rhode Island, College of Nursing, Kingston, RI, Estados Unidos de América.

**Keywords:** Estudio de Validación, Reproducibilidad de los Resultados, Enfermería de Práctica Avanzada, Competencia Profesional, Enfermeras y Enfermeros, Atención Primaria de Salud

## Abstract

**Objetivo:**

evaluar la validez de constructo y la confiabilidad del Instrumento para Evaluación de Competencias del Enfermero de Práctica Avanzada, versión brasileña, en la Atención Primaria de Salud.

**Método:**

estudio metodológico, realizado en 78 unidades de atención primaria de salud de la región sudeste, con 215 enfermeros. Los datos fueron recolectados mediante tres instrumentos: ficha para caracterización de la muestra, versión brasileña del Instrumento para Evaluación de Competencias del Enfermero de Práctica Avanzada y la categoría Intervenciones Terapéuticas de la Escala de Competencia del Enfermero. La validez del constructo fue verificada mediante el análisis factorial confirmatorio y el coeficiente de correlación de Spearman. La confiabilidad fue evaluada con el alfa de Cronbach y la Fiabilidad Compuesta.

**Resultados:**

en el análisis factorial, el modelo convergió hacia un resultado satisfactorio, siendo necesario excluir seis ítems, resultando en un instrumento con 38 ítems distribuidos en ocho dimensiones. En la validez de constructo convergente, se observaron correlaciones positivas, significativas (p<0,0001) y que variaron entre magnitud débil a moderada (r=0,2701 y r=0,397). Se encontraron evidencias satisfactorias mediante el análisis de la Fiabilidad Compuesta (0,78-0,89).

**Conclusión:**

el instrumento demostró evidencias de validez de constructo y consistencia interna. Podrá ser utilizado para apoyar la implementación de estrategias de desarrollo de la práctica avanzada en enfermería.

## Destacados

**(1)** Fomentar la discusión sobre la Práctica Avanzada de Enfermería (PAE) en Brasil.

**(2)** Poner a disposición un instrumento para evaluar la PAE en la Atención Primaria de Salud.

**(3)** Apoyar la implementación de la PAE en Brasil en la Atención Primaria de Salud.

**(4)** La versión brasileña del instrumento puede ser aplicada en diferentes contextos.

**(5)** Estrategia para el fortalecimiento de la fuerza laboral de enfermeros.

## Introducción

La implementación mundial de la Práctica Avanzada de Enfermería (PAE) ha demostrado un impacto positivo y progresivo en la calidad de la atención, los resultados clínicos y la cobertura de los servicios de salud^(1-2)^. Además, se considera una estrategia importante para el reconocimiento^(3)^ y fortalecimiento de la fuerza laboral, incluyendo la cualificación, el reclutamiento y la retención de enfermeros^(4)^, lo que contribuye a la reducción de costos en las instituciones de salud^(1-2,5)^.

Estados Unidos y Canadá fueron los grandes exponentes de esta práctica, y después de la década de los 60, hubo una rápida expansión de la PAE a otros continentes^(6)^. Actualmente, diferentes países se están esforzando por reformular la reglamentación de los alcances de la práctica^(7)^.

La Organización Mundial de la Salud (OMS), junto con la Organización Panamericana de la Salud (OPS), han estado promoviendo la implementación de la PAE en América Latina y el Caribe, especialmente en la Atención Primaria de Salud (APS), dado que es necesario ampliar la atención en regiones remotas y aumentar la cobertura del acceso a los servicios de salud^(4,8)^.

En este sentido, la OPS publicó en 2018 el documento “Ampliación del Rol de los Enfermeros en la Atención Primaria de Salud”^(8)^, y en 2020, la OMS emitió la alerta titulada “Desafíos Urgentes de Salud para la Próxima Década”, resaltando prioridades como el acceso más equitativo a los servicios de salud, la preparación para epidemias, la garantía de acceso a medicamentos y estrategias para invertir en los profesionales que protegen la salud de la población^(9)^.

En Brasil, en 2015 se inició una discusión sobre la “Implementación de la PAE” en el territorio nacional, promovida por el Consejo Federal de Enfermería (Cofen) y la Asociación Brasileña de Enfermería (ABEn)^(10)^, sin embargo, en la actualidad, esta práctica, fundamental para garantizar la calidad de la atención, sigue siendo incipiente^(11)^.

El Enfermero de Práctica Avanzada (EPA) debe contar con una base de conocimientos especializada y competencias para realizar evaluaciones, proponer diagnósticos y prescripciones, implementar programas y planes de atención, y actuar como referente para los usuarios y los servicios de salud^(12)^. Además, es necesario tener un título de maestría, integrar equipos multiprofesionales y contribuir a la gestión de la atención^(8,12)^.

Por lo tanto, los estándares y competencias de los EPA están siendo desarrollados y adaptados para responder a las necesidades de cada país en relación con la formación y la educación continua de los profesionales en los servicios de salud^(13)^. De este modo, los investigadores han estado mapeando un perfil de competencias esenciales para apoyar la definición del alcance de esta práctica^(14)^.

En este contexto, el Inventario para la Evaluación de Competencias en Enfermeras de Práctica Avanzada – IECEPA, desarrollado en España, es considerado un instrumento con evidencias de validez y confiabilidad para evaluar las competencias del EPA, siendo aplicable en cualquier nivel de atención a la salud, con especial énfasis en la APS^(15)^.

En 2022, este instrumento fue adaptado a la cultura brasileña y titulado *Instrumento para* *Avaliação de Competências do Enfermeiro de Prática Avançada* - IECEPA versión brasileña. Su contenido fue evaluado y demostró evidencias de validez^(16)^.

Según la literatura, existen instrumentos para evaluar las competencias de los EPA. Aunque tres han sido construidos y/o adaptados al portugués de Brasil, aún no hay publicaciones científicas sobre la evaluación de la confiabilidad y validez de estos instrumentos^(13-18)^. Se destaca la importancia de utilizar instrumentos con evidencias de confiabilidad y validez para poder evaluar de manera adecuada las competencias de los enfermeros en práctica avanzada.

La aplicación de la herramienta en cuestión en la APS es vasta y prometedora, ya que permitirá mapear las competencias de los EPA e implementar intervenciones que podrían ofrecer beneficios tangibles para la práctica clínica, el desarrollo profesional y el avance de la investigación en el área de la enfermería, pudiendo innovar y reformar los sistemas de salud para responder a los problemas derivados de las necesidades de la población, especialmente debido al aumento de personas con condiciones crónicas en seguimiento en la APS^(2,10)^. Así, el objetivo del presente estudio fue evaluar la validez de constructo y la confiabilidad del Instrumento para Evaluación de Competencias del Enfermero de Práctica Avanzada, versión brasileña, en la Atención Primaria de Salud^(15-16)^.

## Método

### Diseño del estudio

Se trata de un estudio metodológico que tuvo como objetivo analizar la validez de constructo (estructural y prueba de hipótesis) y la confiabilidad (consistencia interna)^(19)^ del IECEPA versión brasileña^(16)^, utilizando los criterios adoptados del *checklist COnsensus-based Standards for the selection of health Measurement INstruments* (COSMIN)^(19)^.

### Lugar de la recolección de datos

Los datos se recolectaron en 78 unidades de APS de dos municipios de la región sudeste. La ciudad “A” tenía 1.194.094 habitantes, 64 Unidades Básicas de Salud (UBS), de las cuales la mayoría incluía la Estrategia de Salud de la Familia (ESF), 291 enfermeros y cinco áreas geográficas, con aproximadamente 200.000 habitantes en cada una. La ciudad “B” tenía 129.193 habitantes, 14 UBS sin la presencia del PSF en el momento de la recolección, y 17 enfermeros, con aproximadamente 9.228 habitantes por cada unidad.

### Período

La etapa de recolección de datos tuvo lugar entre agosto de 2020 y julio de 2021. Entre agosto y octubre de 2020, se realizó de manera presencial y, de octubre de 2020 a julio de 2021, de forma híbrida debido a la pandemia de COVID-19.

### Participantes y criterio de selección

Para estimar el tamaño de la muestra, se adoptó el criterio recomendado internacionalmente para el análisis factorial, el cual considera adecuado un mínimo de 100 participantes y cinco respondientes por cada ítem del instrumento^(19)^, lo que resultó en una muestra de 220 profesionales.

De esta manera, se abordaron 256 enfermeros por conveniencia; sin embargo, se excluyeron 41 (16%) (31 no entregaron el instrumento dentro del plazo acordado y 10 lo entregaron incompleto). Así, la muestra final quedó compuesta por 215 participantes (59 abordados de manera *online* y 156 de manera presencial).

Con base en estos criterios, se seleccionaron por conveniencia a todos los enfermeros activos y se excluyeron aquellos que no completaron uno o más ítems de la versión brasileña del IECEPA.

### Instrumentos utilizados para la recolección de la información

#### Ficha de Caracterización Personal y Profesional

Se recolectaron datos personales (edad, sexo y estado civil) y profesionales (tiempo de experiencia como enfermero, tiempo de experiencia en el área en la que trabajaba y formación).

#### IECEPA versión brasileña ^(16)^

El IECEPA versión brasileña fue diseñado para evaluar las competencias de los enfermeros según los roles y estándares necesarios de la PAE en la atención primaria. Este instrumento cuenta con 44 ítems divididos en ocho dimensiones: Investigación y Práctica Basada en Evidencias: promueve la relación entre investigación, identificación de evidencias científicas y práctica clínica (1.1-1.8); Liderazgo Clínico y Profesional: demuestra liderazgo en la promoción de cuidados de calidad y brinda asesoramiento a otros profesionales de la salud (2.1-2.4); Autonomía Profesional: evalúa la autonomía en el uso de intervenciones farmacológicas y no farmacológicas, diagnóstico clínico y derivación a otros profesionales (3.1-3.8); Relaciones Interprofesionales y Tutoría: refleja la capacidad de colaborar con otros profesionales para mejorar los cuidados al usuario y servir como referente clínico (4.1-4.6); Gestión de Calidad: basado en habilidades para evaluar y promover la calidad y eficacia de los cuidados avanzados, identificando y actuando sobre problemas relacionados con la calidad y seguridad del paciente (5.1-5.4); Gestión de Cuidados: representa la coordinación de la atención en todos los niveles del sistema de salud (6.1-6.6); Enseñanza y Educación Profesional: refleja el rol de educador, promoviendo un entorno propicio para el aprendizaje de usuarios, familiares, enfermeros, estudiantes y otros profesionales de la salud (7.1-7.4) y Promoción de la Salud: se enfoca en la mejora o recuperación de la salud del usuario, independientemente del contexto (8.1-8.4)^(15-16)^.

Estos ítems se evalúan con una escala tipo Likert de cinco puntos, que varía entre nunca (un punto) y siempre (cinco puntos); cuanto mayor es la puntuación, mayor es la frecuencia de uso de la competencia descrita en las actividades profesionales diarias. Para obtener los puntajes de las dimensiones, se calcula el promedio de las respuestas de los participantes^(15-16)^.

#### Escala de Competencia del Enfermero (ECE) ^(20)^

La Escala de Competencia del Enfermero (ECE) es una herramienta que evalúa la frecuencia con que un enfermero utiliza una determinada competencia. Tiene 73 ítems distribuidos en siete categorías. En este estudio, se utilizó únicamente la categoría Intervenciones Terapéuticas (α=0,87), que consta de 10 ítems (39, 40, 41, 42, 43, 44, 45, 46, 47, 48), los cuales reflejan las dimensiones del IECEPA versión brasileña. La escala de respuesta para cada ítem es tipo Likert con cuatro opciones, que varían entre cero (“no se aplica a mi práctica”) y tres (“utilizado muy a menudo”). Cuanto mayor es la puntuación, mayor es la frecuencia con que el profesional utiliza esa competencia en sus actividades profesionales^(20)^. La categoría Intervenciones Terapéuticas de la ECE fue utilizada para evaluar la validez de constructo mediante la prueba de la siguiente hipótesis: cuanto mayor sea el puntaje en las dimensiones de la versión brasileña del IECEPA, mayor será el puntaje en la categoría Intervenciones Terapéuticas de la ECE.

### Recolección de datos

La recolección de datos se llevó a cabo de manera híbrida debido a la situación pandémica, con reclutamiento presencial (*in situ*) y en línea, por medio de correos electrónicos institucionales enviados por las secretarías de salud de las ciudades participantes, siendo realizadas tres rondas de recolección. Los enfermeros fueron abordados en sus lugares de trabajo, se explicó el propósito del estudio y, tras obtener el consentimiento y la firma del Consentimiento Informado (CI), respondieron a los instrumentos impresos.

### Tratamiento y análisis de los datos

Los datos recolectados fueron ingresados en el programa *Excel for Windows/XP* ^®^ y transferidos a los *softwares Statistical Analysis Software* ^®^ versión 9.4 y *Smart Partial Least Squares* (PLS) 3.2.1.^®^ para realizar los análisis. Inicialmente, se utilizaron estadísticas descriptivas, frecuencias absolutas y relativas de las variables categóricas y las medidas de posición (media, mínimo, máximo) y dispersión (desviación estándar) de las variables continuas.

Para analizar las propiedades de medición del IECEPA versión brasileña, se realizó un análisis factorial utilizando modelos de ecuaciones estructurales con PLS^(21)^, en el que las competencias del EPA se consideraron una variable de segundo orden.

En la evaluación del modelo, se calculó la Varianza Media Extraída (VME) para cada dimensión y valores superiores a 0,5 indicaron que el modelo mostró un resultado satisfactorio^(22)^. Se examinaron las cargas cruzadas para determinar si la carga factorial de un ítem era mayor en el factor al que estaba originalmente asignado; además, la validez discriminante del modelo fue verificada mediante el criterio de Fornell-Larcker, que analizó si las raíces cuadradas de la VME eran mayores que las correlaciones entre las dimensiones^(23)^.

Para probar la hipótesis, se utilizó la prueba de Shapiro-Wilk para evaluar la distribución de los datos y el coeficiente de Spearman^(24)^ para la correlación entre los puntajes de la categoría “Intervenciones Terapéuticas” de la ECE con cada una de las dimensiones del IECEPA versión brasileña. Los valores fueron clasificados como débiles (0,1 a 0,29), moderados (0,3 a 0,49) o fuertes (0,5)^(25)^. Se utilizó un nivel de significancia del 5% (p <0,05) en todas las pruebas^(24)^.

La confiabilidad se evaluó mediante la consistencia interna, para lo cual se calcularon el coeficiente alfa de Cronbach y la Fiabilidad Compuesta (FC), considerándose aceptables valores iguales o superiores a 0,70^(24)^.

Para la aceptabilidad, se consideraron satisfactorios valores iguales o superiores al 80%^(26)^.

### Aspectos éticos

Antes de la realización del estudio, se obtuvo la autorización del autor del instrumento original. El proyecto fue aprobado el 26 de marzo de 2020 (formato presencial) y el 21 de octubre de 2020 (formato en línea) por el Comité de Ética en Investigación de la institución, con el Certificado de Presentación para Apreciación Ética n° 23193319.0.0000.5404 (Dictámenes 3.936.606 y 4.351.709, respectivamente), y cumplió con las recomendaciones éticas para investigaciones con seres humanos de acuerdo con la Resolución 466/2012 del Consejo Nacional de Salud.

## Resultados

En cuanto a las características sociodemográficas, de los 215 enfermeros, la edad promedio fue de 39,0 años (ds=7,8), con predominio de mujeres (n=186; 86,5%) y personas casadas (n=124; 57,6%). Con respecto a la caracterización profesional, el tiempo promedio de experiencia en la profesión fue de 12,3 años (ds=7,6) y en el área en la que trabajaban en el momento de la recolección de datos fue de 9,0 años (ds=7,7). La mayoría tenía posgrado en la modalidad de especialización (n=124; 57,6%) y 88 (40,9%) tenían posgrado en el área de Salud Colectiva. En cuanto a la evaluación de la aceptabilidad, de los 256 enfermeros inicialmente abordados, 215 (83,9%) participantes respondieron a todos los ítems del IECEPA versión brasileña para APS, mientras que 31 (12,0%) enfermeros no respondieron, posiblemente debido a la sobrecarga de trabajo causada por la pandemia de COVID-19, y 10 (4,0%) respondieron de forma incompleta.

En los análisis iniciales de la estructura del instrumento, el modelo reveló valores de VME inferiores a 0,50 para cuatro dimensiones, como se puede observar en la Tabla 1.

Después de evaluar la VME, se analizaron las cargas factoriales cruzadas de los ítems del instrumento, lo que llevó a la exclusión de los ítems 1.1 de la dimensión Investigación, 2.3 de la dimensión Liderazgo, 4.6 de la dimensión Relaciones y 6.1 de la dimensión Gestión de Cuidados, ya que presentaron las menores cargas factoriales en sus respectivas dimensiones.

**Tabla 1:** - Modelo inicial de la varianza media extraída, confiabilidad compuesta y alfa de Cronbach de las dimensiones del Instrumento para Evaluación de Competencias del Enfermero de Práctica Avanzada versión brasileña para atención primaria de salud (n = 215). Campinas, SP, Brasil, 2020-2021

Dimensiones del IECEPA* **versión brasileña**		**Modelo Inicial**	
	**VME** ^†^	**Confiabilidad Compuesta**	**Alfa de Cronbach**
1 - Investigación y Práctica Basada en Evidencias	*0,42*	0,85	0,81
2 - Liderazgo Clínico y Profesional	*0,47*	0,78	*0,64*
3 - Autonomía Profesional	0,51	0,89	0,86
4 - Relaciones Interprofesionales y Tutoría	*0,41*	0,81	0,71
5 - Gestión de Calidad	0,56	0,83	0,74
6 - Gestión de Cuidados	*0,47*	0,84	0,77
7 - Enseñanza y Educación Profesional	0,62	0,87	0,79
8 - Promoción de la Salud	0,54	0,82	0,71

*IECEPA = Instrumento para Evaluación de Competencias del Enfermero de Práctica Avanzada versión brasileña; ^†^VME = Varianza Media Extraída

A pesar de la exclusión de cuatro ítems, la VME de las dimensiones Investigación y Relaciones no alcanzó el valor establecido (0,45 y 0,46, respectivamente), por lo que se excluyeron los ítems 1.2 de la dimensión Investigación y 4.1 de la dimensión Relaciones. La Tabla 2 presenta los valores alcanzados para la VME, CC y alfa de Cronbach de cada dimensión del IECEPA - versión brasileña, tras la exclusión de los seis ítems (1.1, 1.2, 2.3, 4.1, 4.6 y 6.1).

**Tabla 2:** - Modelo final de la varianza media extraída, confiabilidad compuesta y alfa de Cronbach de las dimensiones del Instrumento para Evaluación de Competencias del Enfermero de Práctica Avanzada versión brasileña para atención primaria de salud (n = 215). Campinas, SP, Brasil, 2020-2021

Dimensiones del IECEPA* **versión brasileña**		**Modelo Final**	
	**VME** ^†^	**Confiabilidad Compuesta**	**Alfa de Cronbach**
1 - Investigación y Práctica Basada en Evidencias	*0,48*	0,85	0,79
2 - Liderazgo Clínico y Profesional	0,54	0,78	*0,57*
3 - Autonomía Profesional	0,51	0,89	0,86
4 - Relaciones Interprofesionales	0,52	0,81	0,68
5 - Gestión de Calidad	0,56	0,83	0,74
6 - Gestión de Cuidados	0,51	0,84	0,76
7 - Enseñanza y Educación Profesional	0,62	0,87	0,79
8 - Promoción de la Salud	0,55	0,82	0,71

*IECEPA = Instrumento para Evaluación de Competencias del Enfermero de Práctica Avanzada versión brasileña; ^†^VME = Varianza Media Extraída

Después de la exclusión de los ítems, el modelo final mostró que el valor de la VME de la dimensión Investigación no alcanzó el valor establecido. En este sentido, el ítem con menor carga factorial fue el ítem 1.3 “Identifico las prioridades de investigación en mi área de práctica profesional” (0,61), que tuvo una CC y un alfa de Cronbach por encima del valor establecido. Ante esto, las investigadoras decidieron consultar al autor del instrumento original antes de excluir este ítem. El autor recomendó que el ítem fuera mantenido debido a su importancia conceptual, a pesar de que la VME de esta dimensión estuviera por debajo de lo establecido.

Las cargas factoriales de los ítems en sus respectivas dimensiones y las cargas factoriales cruzadas se presentan en la Tabla 3.

**Tabla 3:** - Cargas factoriales de los ítems en sus respectivos constructos (en destaque) y cargas factoriales cruzadas (n = 215). Campinas, SP, Brasil, 2020-2021

**Ítem**	D1*	**D2** ^†^	**D3** ^‡^	**D4** ^§^	**D5** ^||^	**D6** ^¶^	D7**	**D8** ^††^
1.3	*0,61*	0,26	0,05	0,10	0,31	0,22	0,05	0,03
1.4	*0,65*	0,29	0,37	0,24	0,25	0,28	0,09	0,36
1.5	*0,72*	0,37	0,21	0,28	0,32	0,31	0,24	0,24
1.6	*0,75*	0,30	0,19	0,24	0,39	0,31	0,15	0,18
1.7	*0,73*	0,31	0,13	0,11	0,38	0,22	0,05	0,16
1.8	*0,69*	0,41	0,20	0,31	0,32	0,30	0,17	0,26
2.1	0,29	*0,83*	0,23	0,29	0,32	0,24	0,16	0,28
2.2	0,35	*0,77*	0,08	0,23	0,35	0,17	0,10	0,20
2.4	0,44	*0,59*	0,16	0,14	0,26	0,04	0,16	0,12
3.1	0,10	0,21	*0,62*	0,18	-0,01	0,09	0,23	0,37
3.2	0,18	0,21	*0,75*	0,29	0,19	0,23	0,17	0,37
3.3	0,15	0,19	*0,73*	0,25	0,21	0,22	0,25	0,46
3.4	0,36	0,21	*0,74*	0,29	0,25	0,30	0,21	0,48
3.5	0,26	0,16	*0,79*	0,37	0,16	0,40	0,24	0,47
3.6	0,19	0,09	*0,73*	0,23	0,17	0,23	0,14	0,38
3.7	0,10	0,04	*0,73*	0,28	0,04	0,32	0,22	0,46
3.8	0,27	0,14	*0,61*	0,29	0,17	0,24	0,14	0,34
4.2	0,33	0,22	0,27	*0,71*	0,31	0,31	0,22	0,19
4.3	0,19	0,20	0,27	*0,79*	0,32	0,35	0,32	0,33
4.4	0,19	0,17	0,34	*0,64*	0,18	0,27	0,22	0,28
4.5	0,23	0,30	0,24	*0,72*	0,33	0,35	0,23	0,19
5.1	0,28	0,20	0,07	0,27	*0,69*	0,32	0,11	0,19
5.2	0,32	0,30	0,23	0,33	*0,79*	0,40	0,23	0,26
5.3	0,43	0,37	0,28	0,35	*0,81*	0,45	0,17	0,31
5.4	0,36	0,36	0,00	0,23	*0,69*	0,30	0,15	0,19
6.2	0,21	0,14	0,22	0,35	0,38	*0,64*	0,27	0,27
6.3	0,24	0,21	0,33	0,34	0,21	*0,70*	0,25	0,44
6.4	0,36	0,16	0,30	0,34	0,40	*0,83*	0,27	0,42
6.5	0,33	0,16	0,23	0,28	0,41	*0,73*	0,33	0,36
6.6	0,28	0,11	0,22	0,30	0,39	*0,68*	0,42	0,36
7.1	0,14	0,12	0,26	0,28	0,14	0,35	*0,80*	0,35
7.2	0,10	0,16	0,22	0,25	0,18	0,29	*0,79*	0,38
7.3	0,23	0,17	0,19	0,32	0,27	0,42	*0,80*	0,38
7.4	0,12	0,16	0,20	0,23	0,11	0,28	*0,76*	0,33
8.1	0,30	0,15	0,31	0,29	0,32	0,37	0,26	*0,55*
8.2	0,23	0,19	0,44	0,23	0,23	0,31	0,30	*0,74*
8.3	0,17	0,20	0,52	0,29	0,15	0,36	0,33	*0,80*
8.4	0,25	0,27	0,45	0,23	0,28	0,48	0,45	*0,83*

*D1 = Dimensión 1 (Investigación y Práctica Basada en Evidencias); ^†^D2 = Dimensión 2 (Liderazgo Clínico y Profesional); ^‡^D3 = Dimensión 3 (Autonomía Profesional); ^§^D4 = Dimensión 4 (Relaciones Interprofesionales); ^||^D5 = Dimensión 5 (Gestión de Calidad); ^¶^D6 = Dimensión 6 (Gestión de Cuidados); **D7 = Dimensión 7 (Enseñanza y Educación Profesional); ^††^D8 = Dimensión 8 (Promoción de la Salud)

Para evaluar la validez discriminante (Tabla 4), se verificaron las raíces cuadradas de las VME, que fueron más altas en el factor en el que fueron inicialmente asignadas.

**Tabla 4:** - Validez discriminante del modelo factorial, según el criterio de Fornell-Larcker del Instrumento para Evaluación de Competencias del Enfermero de Práctica Avanzada – versión brasileña (n = 215). Campinas, SP, Brasil, 2020-2021

Dimensiones del IECEPA* **versión brasileña**	**1**	**2**	**3**	**4**	**5**	**6**	**7**	**8**
1 - Investigación y Práctica Basada en Evidencias	*0,69*							
2 - Liderazgo Clínico y Profesional	0,47	*0,74*						
3 - Autonomía Profesional	0,29	0,22	*0,71*					
4 - Relaciones Interprofesionales	0,33	0,31	0,39	*0,72*				
5 - Gestión de Calidad	0,47	0,42	0,22	0,40	*0,75*			
6 - Gestión de Cuidados	0,40	022	0,37	0,45	0,50	*0,72*		
7 - Enseñanza y Educación Profesional	0,19	0,19	0,28	0,35	0,23	0,43	*0,79*	
8 - Promoción de la Salud	0,32	0,28	0,59	0,35	0,33	0,52	0,48	*0,74*

*IECEPA = Instrumento para Evaluación de Competencias del Enfermero de Práctica Avanzada versión brasileña

Para la evaluación de la validez de constructo convergente, se probaron las correlaciones entre cada dimensión del IECEPA versión brasileña, y la categoría Intervenciones Terapéuticas de la ECE (Tabla 5).

**Tabla 5:** - Correlación entre las dimensiones de la versión brasileña del Instrumento para Evaluación de Competencias del Enfermero de Práctica Avanzada versión brasileña y la categoría Intervenciones Terapéuticas de la escala de competencia del enfermero (n = 215). Campinas, SP, Brasil, 2020-2021

Dimensiones del IECEPA* **versión brasileña**	**Intervenciones Terapéuticas de la ECE** ^†^
1 - Investigación y Práctica Basada en Evidencias	0,3888 *<0,0001* ^‡^
2 - Liderazgo Clínico y Profesional	0,3508 *<0,0001* ^‡^
3 - Autonomía Profesional	0,2701 *<0,0001* ^‡^
4 - Relaciones Interprofesionales	0,3555 *<0,0001* ^‡^
5 - Gestión de Calidad	0,3555 *<0,0001* ^‡^
6 - Gestión de Cuidados	0,3972 *<0,0001* ^‡^
7 - Enseñanza y Educación Profesional	0,3419 *<0,0001* ^‡^
8 - Promoción de la Salud	0,3049 *<0,0001* ^‡^

*IECEPA = Instrumento para Evaluación de Competencias del Enfermero de Práctica Avanzada versión brasileña; ^†^ECE = Escala de Competencia del Enfermero; ^‡^p-valor = obtenido mediante el coeficiente de correlación de Spearman

## Discusión

Este estudio tuvo como objetivo presentar la evaluación de la validez de constructo y confiabilidad de la versión brasileña del IECEPA en la APS. Según los investigadores, las competencias del EPA deben fomentarse ampliamente, dada la necesidad del desarrollo de habilidades complejas, tanto en la práctica clínica como en las acciones de gestión, educación e investigación^(11,13)^.

Para ello, los instrumentos de medición deben comprender un análisis riguroso de los constructos que pretenden medir para ser utilizados de manera segura en la práctica clínica y de investigación^(27)^. El uso de instrumentos validados para mapear las competencias de la PAE es necesario para impulsar la implementación y la mejora del alcance de esta práctica^(11)^, ya que los estudios muestran que la presencia del EPA en la APS amplía el acceso de los usuarios a los servicios de salud, al tiempo que mejora la capacidad de respuesta de los sistemas de salud frente a desafíos como cambios socioeconómicos, epidemiológicos y climáticos, mediante políticas y acciones de salud actualizadas^(1-2,10)^.

Los datos obtenidos en la presente investigación sugieren que la aceptabilidad del instrumento por parte del público objetivo fue satisfactoria^(26)^, lo que se reflejó en la aceptación del instrumento por parte de los encuestados, ya que la mayoría de los participantes respondieron a todos los ítems. Esta aceptabilidad está en consonancia con otros estudios^(28-29)^.

En cuanto a las propiedades de medición, los resultados demostraron que en la evaluación de la consistencia interna, el alfa de Cronbach de las dimensiones (0,57 - 0,86) fue inferior al del estudio original (0,81 - 0,92)^(15)^. Estas diferencias pueden estar relacionadas con las características de la muestra participante, el contexto y el momento pandémico^(27)^. Sin embargo, la CC, una medida más robusta de la consistencia interna, evidenció una confiabilidad satisfactoria de las dimensiones de la versión brasileña del instrumento^(17)^.

En cuanto a la validez de constructo estructural, se verificó que, en el análisis factorial confirmatorio, se excluyeron seis ítems de cuatro dimensiones para mejorar el ajuste de la VME. En la Dimensión 1 - Investigación, se detectó que, incluso con la exclusión de dos ítems, la VME mostró un valor inferior al establecido. Una posible explicación para este resultado es que esta dimensión no sea tan utilizada en la práctica de los enfermeros participantes de esta investigación, debido a la falta de inversiones en investigación, la alta demanda de atención y/o la falta de apoyo de la gestión^(7)^. Estos datos brindan una oportunidad para discutir la incorporación de la PAE como política pública que respalde a los gestores en diversas áreas, incluida la implementación de la práctica basada en evidencias en el contexto de la APS, con el fin de mejorar aún más la calidad de los servicios de salud y la eficacia de las intervenciones para la población atendida^(7,10-11)^.

En relación al ítem 4.6, se observó que era el único que hacía mención a la palabra “tutoría”. Esto motivó una consulta adicional con especialistas, quienes estuvieron de acuerdo en eliminar la palabra “tutoría” del nombre de la cuarta dimensión, que pasó a llamarse Relaciones Interprofesionales. Algunos estudios también presentaron estas modificaciones en la estructura del instrumento para hacerlo más adecuado a la investigación^(11,28-30)^.

Otro aspecto importante a destacar es que la validez discriminante del modelo fue analizada mediante las cargas cruzadas y el criterio de Fornell-Larcker, evidenciando que los constructos eran independientes y que los ítems eran precisos para medir las dimensiones en las que fueron inicialmente asignados^(22)^.

Con el fin de evaluar la validez de constructo estructural, se utilizó un modelo de ecuaciones estructurales con el método PLS de estimación, que tiene como ventajas la capacidad de construir modelos más complejos y la ausencia del supuesto de que las variables incluidas en el análisis presenten distribución normal^(20)^. Aunque se trata de una metodología relativamente nueva en la literatura científica en comparación con el método tradicional de análisis de covarianza (CB-SEM), el PLS ha sido una opción valiosa en diversas investigaciones^(28-30)^.

Al finalizar los análisis, se constató que la versión brasileña aplicada en la APS tuvo su estructura modificada en relación con la versión original^(15)^, pasando a tener ocho dimensiones y 38 ítems. Esta variación en la estructura del instrumento es una estrategia para mejorar las propiedades de medición y considerar las diferencias culturales entre los países^(31)^. Además, los investigadores se refieren al uso de instrumentos internacionales para evaluar las competencias de los EPA y describen a los enfermeros como aquellos que poseen competencia clínica para actuar en la APS^(1-2,10)^.

En cuanto a los resultados del análisis de validez de constructo convergente, se confirmó la hipótesis formulada, demostrando correlaciones positivas y significativas entre todas las dimensiones de la versión brasileña del IECEPA y la categoría Intervenciones Terapéuticas de la ECE. En siete subescalas, la magnitud de la correlación fue considerada moderada, lo que sugiere que el aumento de la competencia de PAE está asociado con una mayor capacidad para realizar intervenciones terapéuticas en el día a día. Estudios^(28,30)^ corroboran resultados similares respecto a la misma fuerza de correlación.

En vista de ello, la implementación del IECEPA versión brasileña en la APS proporcionará un aporte a la literatura nacional, colaborando en la revisión de proyectos pedagógicos, la implementación de cursos de posgrado y el desarrollo de competencias necesarias para la actuación ampliada de los enfermeros. Además, los resultados de estos estudios pueden ser útiles para el desarrollo de políticas públicas orientadas a la mejora de la calidad de la atención de salud brindada a la población brasileña.

En resumen, la práctica del enfermero en la APS puede mejorarse con la implementación de modelos de EPA, ofreciendo ventajas como la ampliación del acceso y la atención integral, pero también enfrentando desafíos como la resistencia profesional y las barreras legislativas. El éxito de estos modelos depende de inversiones en capacitación, supervisión adecuada y superación de obstáculos regulatorios^(10,17)^.

Aunque existen limitaciones en la aplicación de medidas de autoevaluación, que incluyen la exclusión de casos omisos, el sesgo de memoria, la deseabilidad social y la cognición del entendimiento, este método es constantemente utilizado por los investigadores debido a su beneficio económico, las reflexiones del pasado y la practicidad.

En cuanto al 16% de los datos faltantes, se optó por no imputar la información ausente o incompleta, con el fin de asegurar la confiabilidad y validez de los análisis^(32)^, promoviendo una investigación científica sólida, ya que las técnicas de imputación pueden introducir datos potencialmente imprecisos, causar sesgos o distorsionar las propiedades estadísticas.

El uso de un enfoque híbrido para la recolección de datos presenta diversas ventajas significativas, que incluyen la flexibilidad metodológica, permitiendo la combinación de diferentes métodos de recolección. Esta flexibilidad adaptativa es especialmente relevante en contextos donde restricciones físicas, como las impuestas por la pandemia de COVID-19, pueden impactar la realización de entrevistas presenciales y enfoques convencionales cara a cara^(33)^.

A pesar de que esta investigación abarcó todos los servicios de APS de las dos ciudades, no fue posible obtener el retorno deseado, lo que limita la generalización de los resultados. Además, algunos enfermeros que podrían cumplir con los criterios de definición de PAE pueden no haber sido alcanzados debido a la estrategia de muestreo por conveniencia y a las condiciones derivadas de la pandemia. Se resalta la necesidad de realizar más estudios en diferentes contextos y poblaciones para confirmar las propiedades del instrumento, además de investigaciones transversales, correlacionales y ensayos clínicos. No obstante, se destaca que la validación de la versión brasileña del IECEPA fue rigurosa en sus fases metodológicas y en el análisis estadístico.

## Conclusión

La versión brasileña del IECEPA demostró evidencias de validez de constructo y consistencia interna en la muestra estudiada. Por lo tanto, las contribuciones de este estudio incluyen el avance en la validación de instrumentos de evaluación, el reconocimiento del rol avanzado de los enfermeros y la necesidad urgente de mejorar la formación, además de fomentar la investigación y la práctica basadas en evidencias. Asimismo, resalta la mejora del acceso y la calidad de la atención de salud, así como el apoyo al desarrollo de políticas de salud más eficaces en la APS.

Se destaca la necesidad de negociar la ampliación de la práctica y la creación de legislaciones que respalden al profesional, garantizando que los enfermeros puedan actuar integralmente en la atención de acuerdo con su preparación profesional, contribuyendo así al cumplimiento de las políticas de salud. Su futura aplicación permitirá el mapeo y la implementación de estrategias para el desarrollo de EPA en la APS.

# Translation: Avaliação de competências do enfermeiro de prática avançada: validação de um instrumento para atenção primária^*^

**DOI:** 10.1590/1518-8345.6958.4450

**Authors:** Flávia Carvalho Pena Dias, Thelen Daiana Mendonça Ferreira, Maria Silvia Teixeira Giacomasso Vergílio, Pedro Sastre-Fullana, Thaís Moreira São-João, Renata Cristina Gasparino

**Affiliations:** 1 Universidade Estadual de Campinas, Faculdade de Enfermagem, Campinas, SP, Brasil.; 2 Bolsista da Coordenação de Aperfeiçoamento de Pessoal de Nível Superior (CAPES), Brasil.; 3 Universidade Estadual de Campinas, Grupo de Estudos e Pesquisa em Tecnologias para Gestão em Enfermagem (GEPTGE), Campinas, SP, Brasil.; 4 Hospital Universitari Son Espases, Palma de Mallorca, Illes Balears, PM, Espanha.; 5 University of Rhode Island, College of Nursing, Kingston, RI, Estados Unidos da América.

**Keywords:** Estudo de Validação, Reprodutibilidade dos Testes, Prática Avançada de Enfermagem, Competência Profissional, Enfermeiras e Enfermeiros, Atenção Primária à Saúde

## Abstract

**Objetivo:**

avaliar a validade de construto e a confiabilidade do Instrumento para Avaliação de Competências do Enfermeiro de Prática Avançada, versão brasileira, na Atenção Primária à Saúde.

**Método:**

estudo metodológico, realizado em 78 unidades da atenção primaria à saúde da região sudeste, com 215 enfermeiros. Os dados foram coletados por meio de três instrumentos: ficha para caracterização da amostra, versão brasileira do Instrumento para Avaliação de Competências do Enfermeiro de Prática Avançada e categoria Intervenções Terapêuticas da Escala de Competência do Enfermeiro. A validade do construto foi verificada por meio da análise fatorial confirmatória e do coeficiente de correlação de Spearman. A confiabilidade foi avaliada pelo alfa de Cronbach e Confiabilidade Composta.

**Resultados:**

na análise fatorial, o modelo convergiu para um resultado satisfatório, tendo sido necessário excluir seis itens, resultando em um instrumento com 38 itens distribuídos em oito dimensões. Na validade de construto convergente, foram observadas correlações positivas, significantes (p<0,0001) e variaram entre fraca a moderada magnitude (r=0,2701 e r=0,397). Foram encontradas evidências satisfatórias por meio da análise da Confiabilidade Composta (0,78-0,89).

**Conclusão:**

o instrumento demonstrou evidências de validade de construto e consistência interna. Poderá ser utilizado para auxiliar na implementação de estratégias de desenvolvimento da prática avançada na enfermagem.

## Destaques

**(1)** Fomentar a discussão sobre a Prática Avançada de Enfermagem (PAE) no Brasil.

**(2)** Disponibilizar um instrumento para avaliar a PAE na Atenção Primária à Saúde.

**(3)** Auxiliar na implementação da PAE no Brasil na Atenção Primária à Saúde.

**(4)** A versão brasileira do instrumento pode ser aplicada em diferentes contextos.

**(5)** Estratégia para o fortalecimento da força de trabalho de enfermeiros.

## Introdução

A implementação mundial da Prática Avançada de Enfermagem (PAE) tem demonstrado impacto positivo e progressivo na qualidade do atendimento, resultados clínicos e cobertura dos serviços de saúde^(1-2)^. Além disso, é vista como uma importante estratégia para o reconhecimento^(3)^ e fortalecimento da força de trabalho, incluindo qualificação, recrutamento e retenção de enfermeiros^(4)^, o que contribui para a redução de custos das instituições de saúde^(1-2,5)^.

Os Estados Unidos e o Canadá foram os grandes expoentes dessa prática e após a década de 60, houve rápida expansão da PAE para outros continentes^(6)^. Atualmente, diferentes países vêm se empenhando para reformular a regulamentação dos escopos da prática^(7)^.

A Organização Mundial de Saúde (OMS), junto com a Organização Pan-Americana da Saúde (OPAS), vêm fomentando a implementação da PAE na América Latina e Caribe, especialmente na Atenção Primária à Saúde (APS), tendo em vista a necessidade de ampliar o atendimento em regiões remotas e o aumento da cobertura do acesso aos serviços de saúde^(4,8)^.

Neste âmbito, a OPAS publicou em 2018 o documento “Ampliação do Papel dos Enfermeiros na Atenção Primária à Saúde”^(8)^ e, em 2020, a OMS publicou o alerta intitulado “Desafios Urgentes de Saúde para a Próxima Década” ressaltando prioridades, como o acesso mais justo aos serviços de saúde, preparo para epidemias, garantia de acesso a medicamentos e estratégias para investimentos nos profissionais que protegem a saúde da população^(9)^.

No Brasil iniciou-se, em 2015, uma discussão sobre a “Implementação da PAE” em território nacional, promovida pelo Conselho Federal de Enfermagem (Cofen) e pela Associação Brasileira de Enfermagem (ABEn)^(10)^, porém, nos dias atuais, tal prática, fundamental para a garantia da qualidade da assistência, é ainda incipiente^(11)^.

O Enfermeiro de Prática Avançada (EPA) deve possuir uma base de conhecimentos especializada e competências para realizar avaliações, propor diagnósticos e prescrições, implementar programas e planos de cuidados e atuar como referência para usuários e serviços de saúde^(12)^. Além disso, é necessário ter título de mestrado, integrar equipes multiprofissionais e contribuir para a gestão da assistência^(8,12)^.

Portanto, os padrões e competências dos EPA estão sendo desenvolvidos e adaptados para atender às necessidades de cada país em relação à formação e à educação permanente dos profissionais nos serviços de saúde^(13)^. Sendo assim, pesquisadores vêm mapeando um perfil de competências essenciais para apoiar a definição do escopo desta prática^(14)^.

Nesse contexto, o *Inventario para la Evaluación de Competencias en Enfermeras de* Práctica Avanzada – IECEPA, desenvolvido na Espanha, é considerado um instrumento com evidências de validade e confiabilidade para avaliar as competências do EPA, sendo aplicável em qualquer nível de atenção à saúde, com destaque para a APS^(15)^.

Em 2022, esse instrumento foi adaptado para a cultura brasileira e intitulado Instrumento para Avaliação de Competências do Enfermeiro de Prática Avançada - IECEPA versão brasileira. O seu conteúdo foi avaliado e demonstrou evidências de validade^(16)^.

Segundo a literatura, há instrumentos para avaliar as competências dos EPA. Embora três tenham sido construídos e/ou adaptados para o português do Brasil, ainda não há publicações científicas sobre a avaliação da confiabilidade e validade desses instrumentos^(13-18)^. Ressalta-se a importância da utilização de instrumentos com evidências de confiabilidade e da validade para que se possa avaliar de forma apropriada as competências dos enfermeiros em prática avançada.

A aplicação da ferramenta em questão na APS é vasta e promissora, pois permitirá mapear as competências dos EPA e implementar intervenções que poderão oferecer benefícios tangíveis para a prática clínica, desenvolvimento profissional e avanço da pesquisa na área da enfermagem, podendo inovar e reformar sistemas de saúde para responder aos problemas decorrentes das necessidades da população, especialmente em função do aumento de pessoas com condições crônicas em acompanhamento na APS^(2,10)^. Assim, o objetivo do presente estudo foi avaliar a validade de construto e a confiabilidade do Instrumento para Avaliação de Competências do Enfermeiro de Prática Avançada, versão brasileira, na Atenção Primária à Saúde^(15-16)^.

## Método

### Delineamento do estudo

Trata-se de estudo metodológico que visou analisar a validade de construto (estrutural e teste de hipóteses) e a confiabilidade (consistência interna)^(19)^ do IECEPA - versão brasileira^(16)^, usando os critérios adotados do *checklist* *COnsensus-based Standards for the selection of health Measurement INstruments* (COSMIN)^(19)^.

### Local da coleta de dados

Os dados foram coletados em 78 unidades de APS de dois municípios da região sudeste. A cidade “A” possuía 1.194.094 habitantes, 64 Unidades Básicas de Saúde (UBS), das quais a maioria contempla a Estratégia de Saúde da Família (ESF), 291 enfermeiros e cinco áreas geográficas, com aproximadamente 200.000 habitantes em cada uma. A cidade “B” possuía 129.193 habitantes, 14 UBS sem a presença da ESF na época da coleta e 17 enfermeiros, com cerca de 9.228 habitantes para cada unidade.

### Período

A etapa da coleta de dados se deu entre agosto de 2020 e julho de 2021, sendo que entre agosto e outubro de 2020 ocorreu de forma presencial, e de outubro de 2020 a julho de 2021, de forma híbrida, em função da pandemia de COVID-19.

### Participantes e critério de seleção

Para estimar o tamanho amostral, adotou-se o critério recomendado internacionalmente para a análise fatorial, o qual considera adequado um mínimo de 100 participantes e cinco respondentes para cada item do instrumento^(19)^, resultando em uma amostra de 220 profissionais.

Dessa forma, foram abordados 256 enfermeiros por conveniência, entretanto, 41 (16%) foram excluídos (31 não entregaram o instrumento respondido dentro do prazo combinado e 10 entregaram o instrumento respondido de forma incompleta). Assim, a amostra final foi composta por 215 participantes (59 abordados de maneira *online* e 156 de maneira presencial).

Com base nesses critérios, foram selecionados por conveniência todos os enfermeiros ativos e excluídos aqueles que não preencheram um ou mais itens da versão brasileira do IECEPA.

### Instrumentos utilizados para a coleta das informações

#### Ficha de Caracterização Pessoal e Profissional

Foram coletados dados pessoais (idade, sexo e estado civil) e profissionais (tempo de experiência como enfermeiro, tempo de experiência na área em que atuava e formação).

#### IECEPA versão brasileira^(16)^

O IECEPA versão brasileira foi direcionado para avaliar a competência dos enfermeiros segundo os papéis e padrões necessários da PAE na atenção primária. Este instrumento possui 44 itens divididos em oito dimensões: Pesquisa e Prática com Base em Evidências: promove a relação entre pesquisa, identificação de evidências científicas e prática clínica (1.1-1.8); Liderança Clínica e Profissional: demonstração de liderança na promoção de cuidados de qualidade e fornecimento de aconselhamento a outros profissionais de saúde (2.1-2.4); Autonomia Profissional: avaliação da autonomia no uso de intervenções farmacológicas e não farmacológicas, diagnóstico clínico e encaminhamento para outros profissionais (3.1-3.8); Relações Interprofissionais e Tutoria: reflete a capacidade de colaboração com outros profissionais para melhorar os cuidados à pessoa usuária e servir como referência clínica (4.1-4.6); Gestão de Qualidade: pautada em habilidades para avaliar e promover a qualidade e eficácia dos cuidados avançados, identificando e agindo contra problemas relacionados à qualidade e segurança do paciente (5.1-5.4); Gestão de Cuidados: representa a coordenação do atendimento em todos os níveis de atenção do sistema de saúde (6.1-6.6); Ensino e Educação Profissional: reflete o papel de educador, promovendo um ambiente propício para a aprendizagem de pessoas usuárias, familiares, enfermeiros, estudantes e outros profissionais de saúde (7.1-7.4) e Promoção à Saúde: concentra-se na melhoria ou recuperação da saúde da pessoa usuária, independentemente do contexto (8.1-8.4)^(15-16)^.

Esses itens são avaliados por uma escala tipo Likert com cinco pontos, que variam entre nunca (um ponto) e sempre (cinco pontos); quanto maior a pontuação, maior a frequência de utilização da competência descrita nas atividades profissionais diárias. Para obter os escores das dimensões calcula-se a média das pontuações das respostas dos participantes^(15-16)^.

#### Escala de Competência do Enfermeiro (ECE)^(20)^

A Escala de Competência do Enfermeiro (ECE) é uma ferramenta que avalia a frequência com que um enfermeiro utiliza uma determinada competência. Possui 73 itens distribuídos em sete categorias. Neste estudo, foi usada apenas a categoria Intervenções Terapêuticas (α=0,87), que possui 10 itens (39, 40, 41, 42, 43, 44, 45, 46, 47, 48), que refletem as dimensões do IECEPA versão brasileira. A escala de resposta para cada item é do tipo Likert, com quatro opções que variam entre zero (“não se aplica à minha prática”) e três (“usado muito frequentemente”). Quanto maior a pontuação, maior a frequência com que o profissional utiliza aquela competência em suas atividades profissionais^(20)^. A categoria Intervenções Terapêuticas da ECE foi utilizada para avaliar a validade de construto, por meio do teste da seguinte hipótese: quanto maior o escore nas dimensões da versão brasileira do IECEPA, maior o escore da categoria Intervenções Terapêuticas da ECE.

### Coleta de dados

A coleta de dados foi realizada de forma híbrida devido ao momento pandêmico, com recrutamento presencial (*in situ*) e *online,* por meio de *e-mails* institucionais enviados pelas secretarias de saúde das cidades participantes, tendo sido disparadas três ondas de coleta. Os enfermeiros foram abordados em seus locais de trabalho, explicou-se o propósito do estudo e, após obtenção do consentimento e da assinatura no Termo de Consentimento Livre e Esclarecido (TCLE), eles responderam aos instrumentos impressos.

### Tratamento e análise dos dados

Os dados coletados foram digitados no programa *Excel for Windows/XP* ^®^ e transportados para os *softwares* *Statistical Analysis Software* ^®^ versão 9.4 e *Smart Partial Least Squares* (PLS) 3.2.1.^®^ para condução das análises*.* Inicialmente, foram utilizadas estatísticas descritivas, frequências absolutas e relativas das variáveis categóricas e as medidas de posição (média, mínimo, máximo) e dispersão (desvio-padrão) das variáveis contínuas.

Para analisar as propriedades de medida do IECEPA versão brasileira, foi realizada uma análise fatorial usando modelos de equações estruturais com o PLS^(21)^, em que as competências do EPA foram consideradas uma variável de segunda ordem.

Na avaliação do modelo, a variância média extraída (VME) foi calculada para cada dimensão e valores acima de 0,5 indicaram que o modelo apresentou um resultado satisfatório^(22)^. As cargas cruzadas foram examinadas para determinar se a carga fatorial de um item era mais alta no fator em que estava originalmente alocado; além disso, a validade discriminante do modelo foi verificada pelo critério de Fornell-Larcker, que analisou se as raízes quadradas da VME eram maiores que as correlações entre as dimensões^(23)^.

Para testar a hipótese, utilizou-se o teste de Shapiro-Wilk para avaliar a distribuição dos dados e o coeficiente de Spearman^(24)^ para correlação entre os escores da categoria “Intervenções Terapêuticas” da ECE, com cada uma das dimensões do IECEPA, versão brasileira. Os valores foram classificados como fracos (0,1 a 0,29), moderados (0,3 a 0,49) ou fortes (0,5)^(25)^. Foi usado nível de significância de 5% (p <0,05) em todos os testes^(24)^.

A confiabilidade foi avaliada por meio da consistência interna e, para isso, foram calculados o coeficiente alfa de Cronbach e a Confiabilidade Composta (CC), em que valores iguais ou superiores a 0,70 foram considerados aceitáveis^(24)^.

Para a aceitabilidade, foram considerados satisfatórios valores iguais ou superiores a 80%^(26)^.

### Aspectos éticos

Anteriormente à realização do estudo, foi obtida autorização do autor do instrumento original. O projeto foi aprovado no dia 26 de março de 2020 (formato presencial) e no dia 21 de outubro de 2020 (formato *online*) pelo Comitê de Ética em Pesquisa da instituição, com o Certificado de Apresentação de Apreciação Ética n° 23193319.0.0000.5404 (Pareceres 3.936.606 e 4.351.709, respectivamente), e atendeu às recomendações éticas referentes às pesquisas desenvolvidas com seres humanos de acordo com a Resolução 466/2012 do Conselho Nacional de Saúde.

## Resultados

No que tange às características sociodemográficas, dos 215 enfermeiros, a idade média era de 39,0 anos (dp=7,8), predomínio de mulheres (n=186; 86,5%) e pessoas casadas (n=124; 57,6%). Com relação à caracterização profissional, o tempo médio de experiência na profissão foi de 12,3 anos (dp=7,6) e na área em que ocupava/atuava no momento da coleta de dados foi de 9,0 anos (dp=7,7). A maioria possuía pós-graduação na modalidade especialização (n=124; 57,6%) e 88 (40,9%) tinham pós-graduação na área de Saúde Coletiva. Com relação à avaliação da aceitabilidade, dos 256 enfermeiros inicialmente abordados, 215 (83,9%) participantes responderam a todos os itens do IECEPA versão brasileira para APS, sendo que 31 (12,0%) enfermeiros não responderam, possivelmente devido à sobrecarga de trabalho causada pela pandemia de COVID-19, bem como 10 (4,0%) responderam de forma incompleta.

Nas análises iniciais da estrutura do instrumento, o modelo revelou valores de VME inferiores a 0,50 para quatro dimensões, como pode ser observado na Tabela 1.

**Tabela 1:** - Modelo inicial da variância média extraída, confiabilidade composta e alfa de Cronbach das dimensões do Instrumento para Avaliação de Competências do Enfermeiro de Prática Avançada versão brasileira para atenção primária de saúde (n = 215). Campinas, SP, Brasil, 2020-2021

**Dimensões do IECEPA** ^*^ **versão brasileira**		**Modelo Inicial**	
	**VME** ^†^	**Confiabilidade Composta**	**Alfa de Cronbach**
1 - Pesquisa e Prática com Base em Evidências	*0,42*	0,85	0,81
2 - Liderança Clínica e Profissional	*0,47*	0,78	*0,64*
3 - Autonomia Profissional	0,51	0,89	0,86
4 - Relações Interprofissionais e Tutoria	*0,41*	0,81	0,71
5 - Gestão de Qualidade	0,56	0,83	0,74
6 - Gestão de Cuidados	*0,47*	0,84	0,77
7 - Ensino e Educação Profissional	0,62	0,87	0,79
8 - Promoção da Saúde	0,54	0,82	0,71

*IECEPA = Instrumento para Avaliação de Competências do Enfermeiro de Prática Avançada versão brasileira; ^†^VME = Variância Média Extraída

Após avaliar a VME, foram analisadas as cargas fatoriais cruzadas dos itens do instrumento, o que levou à exclusão dos itens 1.1 da dimensão Pesquisa, 2.3 da dimensão Liderança, 4.6 da dimensão Relações e 6.1 da dimensão Gestão de Cuidados, pois apresentaram as menores cargas fatoriais em suas respectivas dimensões.

Apesar da exclusão de quatro itens, a VME das dimensões Pesquisa e Relações não atingiu o valor estabelecido (0,45 e 0,46, respectivamente) e, por isso, foram excluídos os itens 1.2 da dimensão Pesquisa e 4.1 da dimensão Relações. A Tabela 2 apresenta os valores alcançados para a VME, CC e alfa de Cronbach de cada dimensão do IECEPA versão brasileira, após a exclusão dos seis itens (1.1, 1.2, 2.3, 4.1, 4.6 e 6.1).

**Tabela 2:** - Modelo final da variância média extraída, confiabilidade composta e alfa de Cronbach das dimensões do Instrumento para Avaliação de Competências do Enfermeiro de Prática Avançada versão brasileira para atenção primária de saúde (n = 215). Campinas, SP, Brasil, 2020-2021

**Dimensões do IECEPA** ^*^ **versão brasileira**		**Modelo Final**	
	**VME** ^†^	**Confiabilidade Composta**	**Alfa de Cronbach**
1 - Pesquisa e Prática com Base em Evidências	*0,48*	0,85	0,79
2 - Liderança Clínica e Profissional	0,54	0,78	*0,57*
3 - Autonomia Profissional	0,51	0,89	0,86
4 - Relações Interprofissionais	0,52	0,81	0,68
5 - Gestão de Qualidade	0,56	0,83	0,74
6 - Gestão de Cuidados	0,51	0,84	0,76
7 - Ensino e Educação Profissional	0,62	0,87	0,79
8 - Promoção da Saúde	0,55	0,82	0,71

*IECEPA = Instrumento para Avaliação de Competências do Enfermeiro de Prática Avançada- versão brasileira; ^†^VME = Variância Média Extraída

Após a exclusão dos itens, o modelo final mostrou que o valor da VME da dimensão Pesquisa não atingiu o estabelecido. Sendo assim, o item com menor carga fatorial era o item 1.3 “Identifico as prioridades de pesquisa em minha área de prática profissional” (0,61) e teve uma CC e um alfa de Cronbach acima do estabelecido. Diante disso, as pesquisadoras decidiram consultar o autor do instrumento original antes de excluir esse item. O autor recomendou que o item fosse mantido devido à sua importância conceitual, apesar da VME desta dimensão estar abaixo do estabelecido.

As cargas fatoriais dos itens em suas respectivas dimensões e cargas fatoriais cruzadas foram apresentadas na Tabela 3.

**Tabela 3:** - Cargas fatoriais dos itens em seus respectivos construtos (em destaque) e cargas fatoriais cruzadas (n = 215). Campinas, SP, Brasil, 2020-2021

**Item**	**D1** ^*^	**D2** ^†^	**D3** ^‡^	**D4** ^§^	**D5** ^||^	**D6** ^¶^	**D7** ^**^	**D8** ^††^
1.3	*0,61*	0,26	0,05	0,10	0,31	0,22	0,05	0,03
1.4	*0,65*	0,29	0,37	0,24	0,25	0,28	0,09	0,36
1.5	*0,72*	0,37	0,21	0,28	0,32	0,31	0,24	0,24
1.6	*0,75*	0,30	0,19	0,24	0,39	0,31	0,15	0,18
1.7	*0,73*	0,31	0,13	0,11	0,38	0,22	0,05	0,16
1.8	*0,69*	0,41	0,20	0,31	0,32	0,30	0,17	0,26
2.1	0,29	*0,83*	0,23	0,29	0,32	0,24	0,16	0,28
2.2	0,35	*0,77*	0,08	0,23	0,35	0,17	0,10	0,20
2.4	0,44	*0,59*	0,16	0,14	0,26	0,04	0,16	0,12
3.1	0,10	0,21	*0,62*	0,18	-0,01	0,09	0,23	0,37
3.2	0,18	0,21	*0,75*	0,29	0,19	0,23	0,17	0,37
3.3	0,15	0,19	*0,73*	0,25	0,21	0,22	0,25	0,46
3.4	0,36	0,21	*0,74*	0,29	0,25	0,30	0,21	0,48
3.5	0,26	0,16	*0,79*	0,37	0,16	0,40	0,24	0,47
3.6	0,19	0,09	*0,73*	0,23	0,17	0,23	0,14	0,38
3.7	0,10	0,04	*0,73*	0,28	0,04	0,32	0,22	0,46
3.8	0,27	0,14	*0,61*	0,29	0,17	0,24	0,14	0,34
4.2	0,33	0,22	0,27	*0,71*	0,31	0,31	0,22	0,19
4.3	0,19	0,20	0,27	*0,79*	0,32	0,35	0,32	0,33
4.4	0,19	0,17	0,34	*0,64*	0,18	0,27	0,22	0,28
4.5	0,23	0,30	0,24	*0,72*	0,33	0,35	0,23	0,19
5.1	0,28	0,20	0,07	0,27	*0,69*	0,32	0,11	0,19
5.2	0,32	0,30	0,23	0,33	*0,79*	0,40	0,23	0,26
5.3	0,43	0,37	0,28	0,35	*0,81*	0,45	0,17	0,31
5.4	0,36	0,36	0,00	0,23	*0,69*	0,30	0,15	0,19
6.2	0,21	0,14	0,22	0,35	0,38	*0,64*	0,27	0,27
6.3	0,24	0,21	0,33	0,34	0,21	*0,70*	0,25	0,44
6.4	0,36	0,16	0,30	0,34	0,40	*0,83*	0,27	0,42
6.5	0,33	0,16	0,23	0,28	0,41	*0,73*	0,33	0,36
6.6	0,28	0,11	0,22	0,30	0,39	*0,68*	0,42	0,36
7.1	0,14	0,12	0,26	0,28	0,14	0,35	*0,80*	0,35
7.2	0,10	0,16	0,22	0,25	0,18	0,29	*0,79*	0,38
7.3	0,23	0,17	0,19	0,32	0,27	0,42	*0,80*	0,38
7.4	0,12	0,16	0,20	0,23	0,11	0,28	*0,76*	0,33
8.1	0,30	0,15	0,31	0,29	0,32	0,37	0,26	*0,55*
8.2	0,23	0,19	0,44	0,23	0,23	0,31	0,30	*0,74*
8.3	0,17	0,20	0,52	0,29	0,15	0,36	0,33	*0,80*
8.4	0,25	0,27	0,45	0,23	0,28	0,48	0,45	*0,83*

*D1 = Dimensão 1 (Pesquisa e Prática com Base em Evidências); ^†^D2 = Dimensão 2 (Liderança Clínica e Profissional); ^‡^D3 = Dimensão 3 (Autonomia Profissional); ^§^D4 = Dimensão 4 (Relações Interprofissionais); ^||^D5 = Dimensão 5 (Gestão de Qualidade); ^¶^D6 = Dimensão 6 (Gestão de Cuidados); ^**^D7 = Dimensão 7 (Ensino e Educação Profissional); ^††^D8 = Dimensão 8 (Promoção da Saúde)

Para avaliar a validade discriminante (Tabela 4), foram verificadas as raízes quadradas das VME, mais elevadas no fator em que foram inicialmente alocadas.

**Tabela 4:** - Validade discriminante do modelo fatorial, segundo Critério de Fornell-Larcker do Instrumento para Avaliação de Competências do Enfermeiro de Prática Avançada versão brasileira (n = 215). Campinas, SP, Brasil, 2020-2021

**Dimensões do IECEPA** ^*^ **versão brasileira**	**1**	**2**	**3**	**4**	**5**	**6**	**7**	**8**
1- Pesquisa e Prática em Evidências	*0,69*							
2- Liderança Clínica e Profissional	0,47	*0,74*						
3 - Autonomia Profissional	0,29	0,22	*0,71*					
4 - Relações Interprofissionais	0,33	0,31	0,39	*0,72*				
5 - Gestão de Qualidade	0,47	0,42	0,22	0,40	*0,75*			
6 - Gestão de Cuidados	0,40	022	0,37	0,45	0,50	*0,72*		
7- Ensino e Educação Profissional	0,19	0,19	0,28	0,35	0,23	0,43	*0,79*	
8 - Promoção da Saúde	0,32	0,28	0,59	0,35	0,33	0,52	0,48	*0,74*

*IECEPA = Instrumento para Avaliação de Competências do Enfermeiro de Prática Avançada versão brasileira

Para avaliação da validade de construto convergente, foram testadas as correlações entre cada dimensão do IECEPA, versão brasileira, e a categoria Intervenções Terapêuticas da ECE (Tabela 5).

**Tabela 5:** - Correlação entre as dimensões da versão brasileira do instrumento para avaliação de competências do enfermeiro de prática avançada – versão brasileira e a categoria intervenções terapêuticas da escala de competência do enfermeiro (n = 215). Campinas, SP, Brasil, 2020-2021

**Dimensões do IECEPA** ^*^ **versão brasileira**	**Intervenções Terapêuticas da ECE** ^†^
1 - Pesquisa e Prática com Base em Evidências	0,3888 *<0,0001* ^‡^
2 - Liderança Clínica e Profissional	0,3508 *<0,0001* ^‡^
3 - Autonomia Profissional	0,2701 *<0,0001* ^‡^
4 - Relações Interprofissionais	0,3555 *<0,0001* ^‡^
5 - Gestão de Qualidade	0,3555 *<0,0001* ^‡^
6 - Gestão de Cuidados	0,3972 *<0,0001* ^‡^
7 - Ensino e Educação Profissional	0,3419 *<0,0001* ^‡^
8- Promoção da Saúde	0,3049 *<0,0001* ^‡^

*IECEPA = Instrumento para Avaliação de Competências do Enfermeiro de Prática Avançada- versão brasileira; ^†^ECE = Escala de Competência do Enfermeiro; ^‡^p-valor = obtido por meio do coeficiente de correlação de Spearman

## Discussão

Este estudo teve como objetivo apresentar a avaliação da validade de construto e confiabilidade da versão brasileira do IECEPA na APS. Segundo pesquisadores, as competências do EPA devem ser amplamente fomentadas, dada a necessidade do desenvolvimento de habilidades complexas, tanto na prática clínica como nas ações de gerenciamento, educação e pesquisa^(11,13)^.

Para isso, instrumentos de mensuração devem compreender uma análise rigorosa dos construtos que se propõem a medir para serem utilizados com segurança na prática clínica e de pesquisa^(27)^. A utilização de instrumentos validados para mapear as competências da PAE é necessária para impulsionar a implementação e a melhoria do escopo desta prática^(11)^, pois estudos mostram que a presença do EPA na APS amplia o acesso dos usuários aos serviços de saúde, ao mesmo tempo em que melhora a capacidade de resposta dos sistemas de saúde diante de desafios como mudanças socioeconômicas, epidemiológicas e climáticas, por meio de políticas e ações de saúde atualizadas^(1-2,10)^.

Os dados obtidos na presente pesquisa sugerem que a aceitabilidade do instrumento pelo público-alvo foi satisfatória^(26)^, o que refletiu na aceitação do instrumento pelos respondentes, visto que a maioria dos participantes respondeu todos os itens. Essa aceitabilidade está em consonância com outros estudos^(28-29)^.

Em relação às propriedades de medida, os resultados demonstraram que na avaliação da consistência interna, o alfa de Cronbach das dimensões (0,57 - 0,86) foi inferior ao do estudo original (0,81 - 0,92)^(15)^. Essas alterações podem estar relacionadas às características da amostra participante, ao contexto e ao momento pandêmico^(27)^. Porém, a CC, uma medida mais robusta da consistência interna, evidenciou uma confiabilidade satisfatória das dimensões da versão brasileira do instrumento^(17)^.

Na validade de construto estrutural, verificou-se que, na análise fatorial confirmatória, seis itens de quatro dimensões foram excluídos para melhor ajuste da VME. Na dimensão 1 - Pesquisa, foi detectado que, mesmo com a exclusão de dois itens, a VME demonstrou valor inferior ao estabelecido. Uma provável elucidação para esse resultado é que essa dimensão não seja tão utilizada na prática dos enfermeiros participantes desta pesquisa, devido à falta de investimentos em pesquisa, à alta demanda do cuidado e/ou à ausência de suporte da gestão^(7)^. Esses dados trazem oportunidade de discussão para a incorporação da PAE como política pública que amparem os gestores em inúmeras vertentes, inclusive na implementação da prática baseada em evidências no contexto da APS, com o intuito de melhorar ainda mais a qualidade dos serviços de saúde e a eficácia das intervenções para a população atendida^(7,10-11)^.

Referente ao item 4.6, observou-se que era o único que fazia menção à palavra “tutoria”. Isso motivou uma consulta adicional com especialistas, que concordaram em remover a palavra “tutoria” do nome da quarta dimensão, que passou a ser chamada de Relações Interprofissionais. Alguns estudos apresentaram também essas alterações na estrutura do instrumento para torná-lo mais adequado à pesquisa^(11,28-30)^.

Outro aspecto importante a se destacar é que a validade discriminante do modelo foi analisada por meio das cargas cruzadas e do critério de Fornell-Larcker, evidenciando que os construtos eram independentes e que os itens eram precisos para mensurar as dimensões nas quais foram inicialmente alocados^(22)^.

A fim de avaliar a validade de construto estrutural, foi utilizado um modelo de equações estruturais com o método PLS de estimação, que possui como vantagens a capacidade de construir modelos mais complexos e a ausência do pressuposto que as variáveis incluídas na análise apresentem distribuição normal^(20)^. Embora seja uma metodologia relativamente nova na literatura científica em comparação com o método tradicional de análise de covariância (CB-SEM), o PLS tem sido uma opção valiosa em diversas pesquisas^(28-30)^.

Ao término das análises, constatou-se que a versão brasileira aplicada na APS teve sua estrutura modificada em relação à versão original^(15)^, passando a ter oito dimensões e 38 itens. Essa variação na estrutura do instrumento é uma estratégia para aprimorar as propriedades de medida e considerar as diferenças culturais entre os países^(31)^. Ademais, pesquisadores referem-se à utilização de instrumentos internacionais para avaliar a competência dos EPA e descrevem os enfermeiros como aqueles que possuem competência clínica para atuarem na APS^(1-2,10)^.

Com relação aos resultados da análise de validade de construto convergente, foi confirmada a hipótese formulada, demostrando correlações positivas e significativas entre todas as dimensões da versão brasileira do IECEPA e a categoria Intervenções Terapêuticas da ECE. Em sete subescalas, a magnitude da correlação foi considerada moderada, o que sugere que o aumento da competência de PAE está associado a uma maior capacidade de desempenhar intervenções terapêuticas no cotidiano. Estudos^(28,30)^ corroboram resultados semelhantes referentes à mesma força da correlação.

Em vista disso, a viabilização do IECEPA versão brasileira na APS fornecerá um aporte à literatura nacional, colaborando na revisão de projetos pedagógicos e implementação de cursos de pós-graduação e no desenvolvimento de competências necessárias para a atuação ampliada dos enfermeiros. Além disso, os resultados desses estudos podem ser úteis para o desenvolvimento de políticas públicas na melhoria da qualidade da assistência à saúde oferecida à população brasileira.

Em resumo, a prática do enfermeiro na APS pode ser aprimorada com a implementação de modelos de EPA, oferecendo vantagens como ampliação do acesso e cuidado integral, mas também enfrentando desafios como resistência profissional e barreiras legislativas. O sucesso desses modelos depende de investimentos em capacitação, supervisão adequada e superação de obstáculos regulatórios^(10,17)^.

Embora existam limitações na aplicação das medidas de autoavaliação que incluem a exclusão de casos omissos, o viés de memória, a desejabilidade social e a cognição do entendimento, este método é constantemente utilizado pelos pesquisadores devido ao benefício econômico, reflexões do passado e praticabilidade.

Quanto aos 16% dos dados faltantes, optou-se por não imputar as informações ausentes ou faltantes, com o intuito de assegurar a confiabilidade e validade das análises^(32)^, promovendo uma investigação científica sólida, uma vez que as técnicas de imputação podem introduzir dados potencialmente imprecisos, causar viés ou distorcer as propriedades estatísticas.

A utilização de uma abordagem de coleta de dados híbrida apresenta diversas vantagens significativas, que incluem a flexibilidade metodológica, permitindo a combinação de diferentes métodos de coleta de dados. Essa flexibilidade adaptativa é especialmente relevante em contextos nos quais restrições físicas, como as impostas pela pandemia de COVID-19, podem impactar a condução de entrevistas presenciais e abordagens pessoais convencionais^(33)^.

Apesar desta pesquisa ter abrangido todos os serviços de APS das duas cidades, não foi possível obter o retorno desejado, o que limita a generalização dos resultados. Além disso, alguns enfermeiros que poderiam atender aos critérios de definição de PAE podem não ter sido alcançados, devido à abordagem de pesquisa por conveniência e às condições decorrentes da pandemia.

Ressalta-se que se faz necessário realizar mais estudos em diferentes contextos e populações para confirmar as propriedades do instrumento, além de pesquisas transversais, correlacionais e ensaios clínicos. No entanto, destaca-se que a validação da versão brasileira do IECEPA foi rigorosa em suas fases metodológicas e análise estatística.

## Conclusão

O IECEPA versão brasileira demonstrou evidências de validade de construto e consistência interna na amostra estudada. Portanto, as contribuições deste estudo incluem o avanço na validação de instrumentos de avaliação, o reconhecimento da atuação avançada dos enfermeiros e a necessidade premente de aprimorar a formação além de incentivar a pesquisa e a prática baseadas em evidências. Além disso, salienta a melhoria do acesso e qualidade dos cuidados de saúde, bem como o apoio ao desenvolvimento de políticas de saúde mais eficazes na APS.

Destaca-se a necessidade de negociar a ampliação da prática e a criação de legislações que respaldem o profissional, garantindo que os enfermeiros possam atuar integralmente na assistência de acordo com seu preparo profissional, contribuindo, assim, para o cumprimento das políticas de saúde. Sua aplicação futura permitirá o mapeamento e a implementação de estratégias de desenvolvimento de EPA na APS.
